# Computational Splicing Analysis of Transcriptomic Data Reveals Sulforaphane Modulation of Alternative mRNA Splicing of DNA Repair Genes in Differentiated SH-SY5Y Neurons

**DOI:** 10.3390/ijms26178187

**Published:** 2025-08-23

**Authors:** Maria Lui, Luigi Chiricosta, Renato Iori, Emanuela Mazzon, Aurelio Minuti, Osvaldo Artimagnella

**Affiliations:** 1 IRCCS Centro Neurolesi “Bonino-Pulejo”, Via Provinciale Palermo, Contrada Casazza, 98124 Messina, Italy; 2 Department of Food Quality and Nutrition, Research and Innovation Centre, Fondazione Edmund Mach (FEM), Via E. Mach 1, 38098 San Michele all’Adige, Italy; 3 Department of Innovative Technologies in Medicine & Dentistry, University “G. D’Annunzio” Chieti-Pescara, Via dei Vestini, 31, 66100 Chieti, Italy

**Keywords:** sulforaphane, isothiocyanate, alternative splicing analysis, DNA repair, neuronal cells

## Abstract

Sulforaphane (SFN) is a bioactive compound belonging to the isothiocyanate family, known for its neuroprotective properties. While transcriptomic studies have highlighted SFN’s role in regulating gene expression, its impact on alternative splicing (AS), a key regulatory mechanism in neuronal metabolism, remains underexplored. In this study, we investigated whether SFN pre-treatment influences mRNA splicing patterns in an in vitro neuronal model using retinoic acid (RA)-differentiated SH-SY5Y cells. Using a dedicated RNA-seq-based splicing analysis pipeline, we identified 194 differential alternative splicing events (DASEs) associated with SFN treatment. Gene Ontology enrichment revealed significant over-representation of DNA repair processes. To better understand the functional implications, we integrated in silico predictions of premature stop codons, DASE/miRNA hybridizations, and DASE/RNA-binding protein (RBP) motif occurrences. Our findings suggest that SFN may modulate splicing of key DNA repair genes, contributing to protecting neurons against DNA damage. These preliminary results underscore a novel layer of SFN’s molecular effects and propose it as a valuable adjuvant in physiological conditions to enhance cellular health. Further studies are warranted to dissect the mechanistic underpinnings of SFN-mediated AS and its relevance in DNA-damage-related disorders.

## 1. Introduction

Sulforaphane (SFN, 4-methylsulfinylbutyl isothiocyanate) is an aliphatic compound derived from the hydrolysis of glucoraphanin, a glucosinolate abundantly found in cruciferous vegetables such as broccoli, cauliflower, cabbage, and Brussels sprouts [1]. When these vegetables are cut or chewed, glucoraphanin is converted into the bioactive compound SFN through a hydrolytic reaction catalyzed either by the plant’s own myrosinase enzyme [2] or, alternatively, by bacterial enzymes in the colon [3].

SFN exerts a broad spectrum of biological effects, including antioxidant, anti-inflammatory, anticancer, and cell-protective properties [2,3,4]. Regarding its antioxidant effects, SFN is well-known for its strong ability to activate the nuclear factor erythroid 2-related factor 2 (Nrf2) antioxidant response element (ARE) pathway, a key regulator of cellular defense mechanisms against oxidative stress [5]. In addition, emerging evidence suggests that SFN influences DNA repair mechanisms by activating DNA repair pathways and enhancing the resolution of specific types of DNA damage levels [6]. While most studies to date have focused on in vitro models, SFN has also demonstrated beneficial effects in animal models of neurodegeneration and aging, including improvements in cognitive function and activation of DNA repair mechanisms [7]. Inflammation and immune dysregulation are widely recognized as core physiological disturbances in individuals affected by neurodegenerative diseases. In this context, recent preclinical research has explored the potential neuroprotective effects of SFN, showing encouraging outcomes [8].

In previous work, bioactivated glucoraphanin was shown to enhance neuronal survival, activate the PI3K-AKT signaling pathway, and upregulate genes involved in DNA repair in an in vitro model of Alzheimer’s disease [6]. Animal studies indicate that SFN holds promise as a disease-modifying agent for several prevalent and debilitating central nervous system (CNS) disorders, including Alzheimer’s disease, Parkinson’s disease, epilepsy, and stroke. Central to these disorders are disruptions in key biological pathways—such as oxidative stress, inflammation, and gene regulation—among which alternative splicing (AS) has recently emerged as a vital mechanism [9].

Interestingly, SFN has been reported to regulate AS in a murine model of prostate cancer [10], suggesting that this mechanism delays cancer progression. No data are yet available about SFN’s implications in neuronal AS.

AS is a widespread post-transcriptional mechanism that allows a single pre-mRNA transcript to generate multiple distinct mRNA isoforms, thereby greatly enhancing protein complexity and functional diversity—a mechanism that plays a crucial role in the nervous system [11]. This intricate process is tightly regulated through the interplay of cisregulatory elements and trans-acting factors, and it can be modulated by various cellular signaling pathways [12,13].

AS is essential for tissue development, cellular differentiation, and the regulation of other key cellular pathways such as the DNA damage response (DDR) [14].

AS of pre-mRNA is orchestrated by the spliceosome, a large macromolecular complex made up of five small nuclear RNAs (U1, U2, U4, U5, and U6) and numerous associated proteins, forming small nuclear ribonucleoproteins (snRNPs). This complex, assembled through interactions between cis-acting elements and trans-acting factors, mediates precise RNA–RNA, RNA–protein, and protein–protein interactions. Splicing regulatory elements (SREs), located in exonic or intronic enhancer and silencer regions, influences splicing by recruiting specific trans-acting proteins [15].

RNA-binding proteins (RBPs) orchestrate the assembly of ribonucleoprotein complexes that regulate RNA processing, stability, localization, and translation, thereby influencing virtually all stages of gene expression [9]. Intron retention (IR) represents a distinct form of AS with important regulatory implications: transcripts harboring retained introns often include premature termination codons, marking them for degradation via nonsense-mediated decay (NMD), or possibly generating truncated or non-functional protein isoforms [16]. More intriguingly, several retained introns, especially those located within 3′UTRs, enrich the transcript with additional miRNA binding sites [17]. This not only enhances miRNA-mediated repression but also raises the possibility that intron-retaining transcripts could act as miRNA sponges, modulating the availability of miRNAs and indirectly regulating gene expression [18]. Both IR-coupled NMD and miRNA interactions therefore contribute to fine-tuned post-transcriptional control. Disruption of AS regulation, particularly involving RBPs, has been linked to various diseases, most notably neurodegenerative disorders [9,19]. The proper function of RBPs is essential for maintaining cellular homeostasis, and their dysfunction has been associated with a range of pathological conditions, including neurodegeneration [20]. Nevertheless, the specific effects of SFN on AS remain largely uncharacterized.

This study aims to investigate the impact of SFN on mRNA splicing regulation in retinoic acid (RA)-differentiated SH-SY5Y neuronal cells. To achieve this, an mRNA splicing analysis pipeline was applied to transcriptomic sequencing data to identify differentially alternative splicing events (DASEs). These events were further analyzed for potential regulatory interactions, including DASE/micro-RNA (miRNA) hybridization and DASE/RNA-binding protein (RBP) motif enrichment.

## 2. Results

### 2.1. Sulforaphane Treatment Induces Alternative Splicing Events on RA-Differentiated SH-SY5Y Cells

**Figure 1:**
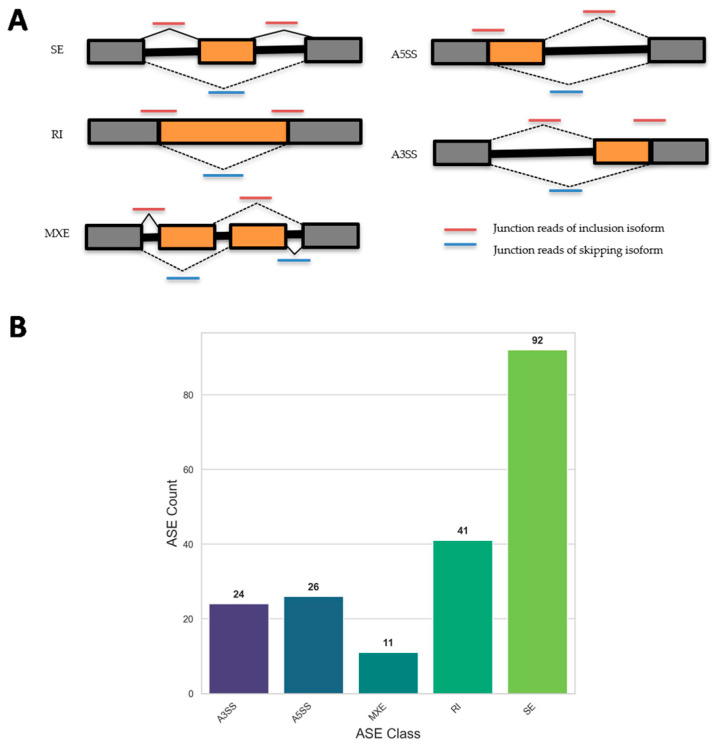
rMATS analysis of DASE identification for SFN5 vs. CTR comparison. (**A**) Schematic illustration of rMATS method for detecting alternative splicing events, adapted from [22], designed to make it easier to interpret effects of alternative splicing events on exon and intron usage, which can otherwise be complex to understand. Five major types of events are shown: A3SS, A5SS, MXE, RI, and SE. In each schematic representation of splicing event, alternatively spliced exons or introns are highlighted in orange, while constitutive flanking exons are shown in gray. Red arcs represent splice junction reads supporting inclusion isoform, whereas blue arcs indicate junction reads supporting skipping isoform. These junction read patterns are used by rMATS to classify splicing events. (**B**) Bar plot showing number of DASEs identified for each event type in SFN5 vs. CTR comparison. Values above each bar indicate total count of events detected per splicing class.

To investigate the impact of SFN on AS processes, we sequenced the transcriptome of RA-differentiated SH-SY5Y treated with 5 µM SFN (SFN5; a concentration that we previously reported to be safe and biologically active [6]) and control (CTR) samples. Their sequenced transcriptomes were used to detect and quantify differential alternative splicing events (DASEs) by the rMATS (replicate Multivariate Analysis of Transcript Splicing) tool [21]. Splicing events included the following: A3SS (alternative 3′ splice site), A5SS (alternative 5′ splice site), MXE (mutually exclusive exon), RI (retained intron), and SE (skipped exon), as schematized in Figure 1A. Specifically, rMATs identified a total of 194 DASEs modulated by SFN5 with respect to CTR (Table S1), which comprised 24 A3SSs, 26 A5SSs, 11 MXEs, 41 RIs, and 92 SEs (Figure 1B). Splicing analysis quality metrics—including coverage, Percent Spliced In (PSI) distribution, and read mapping rate—were evaluated and are presented in Figure S1.

### 2.2. DASEs Regulated by Sulforaphane Encode for a Set of Intercontected Proteins Involved in DNA Repair Mechanisms

**Figure 2:**
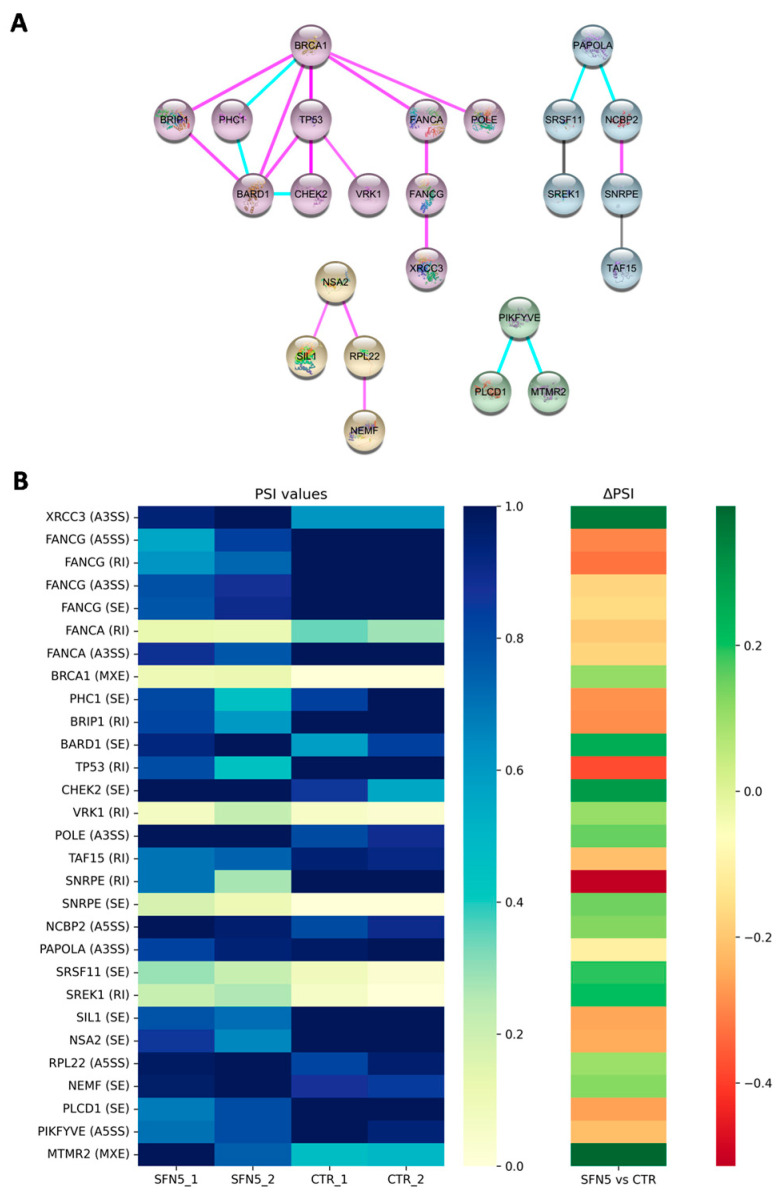
PPI network and its relative heatmap. (**A**) Main clusters of the PPI network made of genes regulated by SFN at the splicing level. Cluster 1 (violet nodes) includes the following: *XRCC3*, *FANCG*, *FANCA*, *BRCA1*, *PHC1*, *BRIP1*, *BARD1*, *TP53*, *CHEK2*, *VRK1*, and *POLE*. Cluster 2 (light blue nodes) comprises the following: *TAF15*, *SNRPE*, *NCBP2*, *PAPOLA*, *SRSF11*, and *SREK1*. Cluster 3 (yellow nodes) contains the following: *SIL1*, *NSA2*, *RPL22*, and *NEMF.* Cluster 4 (green nodes) consists of the following: *PLCD1*, *PIKFYVE*, and *MTMR2.* Pink edges indicate interactions supported by experimental evidence, light blue edges represent interactions curated from established databases, and black edges denote co-expression-based interactions. (**B**) Heatmap of PSI values and ΔPSI values for each DASE detected on the genes belonging to the four identified clusters.

The 194 DASEs were composed of 160 different genes which were further investigated for their function to check whether they were involved in common biological processes or pathways or, in general, exerted similar effects. The protein encoded by these 160 genes made up the nodes of the protein–protein interaction (PPI) network we constructed using interaction data retrieved from the STRING database. We mainly identified four distinct clusters of highly interconnected nodes (Figure 2), representing functional modules, each potentially involved in different processes or pathways. To understand the biological significance of them, we performed a PANTHER Gene Ontology (GO) over-representation analysis (ORA), which provided detailed annotations of GO terms—specifically focusing on biological processes (BPs) associated with each cluster.

The biggest cluster (we named it “Cluster 1”, shown with violet nodes in Figure 2), made by *XRCC3*, *FANCG*, *FANCA*, *BRCA1*, *PHC1*, *BRIP1*, *BARD1*, *TP53*, *CHEK2*, *VRK1*, and *POLE*, resulted in the enrichment of eight different biological processes listed in Table 1. Interestingly, all the enriched biological processes were related to DNA damage response and repair pathways. Cluster 2 (light blue nodes) included *TAF15*, *SNRPE*, *NCBP2*, *PAPOLA*, *SRSF11*, and *SREK1* which resulted in the enrichment of biological processes related to RNA processing and splicing functions (as reported in Table 1). Clusters 3 and 4 are represented in Figure 2 with yellow and green nodes, respectively, and did not significantly enrich any specific biological process.

**Figure 3:**
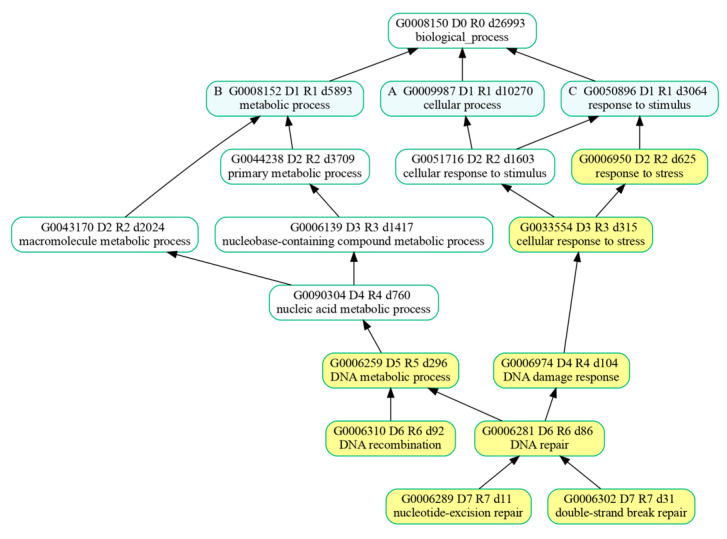
A directed acyclic graph illustrating the biological processes enriched among the genes in Cluster 1 and their hierarchical relationships. Yellow nodes represent the enriched biological processes listed in Table 1, while solid black arrows indicate a formal ‘is a’ relationship between them.

Notably, Cluster 1 emerged as particularly noteworthy because its associated biological processes proved to be highly specific, suggesting a clear connection between splicing events and DNA repair processes (as highlighted in Figure 3).

This evidence determined our choice to focus on this set of 11 genes, which exhibited 15 DASEs in response to SFN treatment compared to the CTR condition. The PSI values for each event, as determined by rMATS, were compared between the SFN5 and CTR groups to pinpoint splicing events with statistically significant differences expressed by ΔPSI values. These 15 DASEs, associated with the 11 genes from Cluster 1, are summarized in Table 2 along with their corresponding ΔPSI values. In Figure S2, we displayed their sashimi plots following same order given in Table 2.

### 2.3. Sulforaphane Downregulates RI Events of DNA Repair DASEs Preventing Non-Functional Proteins

Following SFN treatment, five genes from Cluster 1 exhibited differential splicing characterized by abnormal intron retention. Specifically, *BRIP1*, *FANCG*, *TP53*, and *FANCA* showed negative ΔPSI values, indicating a reduction in intron-retaining isoforms upon SFN treatment. In contrast, *VRK1* was the only gene with a positive ΔPSI, suggesting increased intron retention. These findings suggest that SFN treatment generally seems to reduce the amount of intron-retaining transcripts.

To further deepen the potential functional implications, the retained intronic regions were analyzed for the presence of premature stop codons, which could result in truncated and non-functional proteins. This analysis aimed to establish a mechanistic link between SFN-induced splicing events and potential impacts on gene expression and DNA repair capacity.

Given that there exist three possible reading frames on a single strand (depending on whether the codon starts at the 1st, 2nd, or 3rd nucleotide), for each RI event we manually checked the correct reading frame and reported it in Table 3. Once the correct reading frame had been determined for each RI event, we were able to check for the presence of premature stop codons in the intronic region. The nucleotide position of the first premature stop codon encountered is reported in Table 3 and Table S2, along with the total number of premature stop codons present in the intronic region of each RI event.

**Figure 4:**
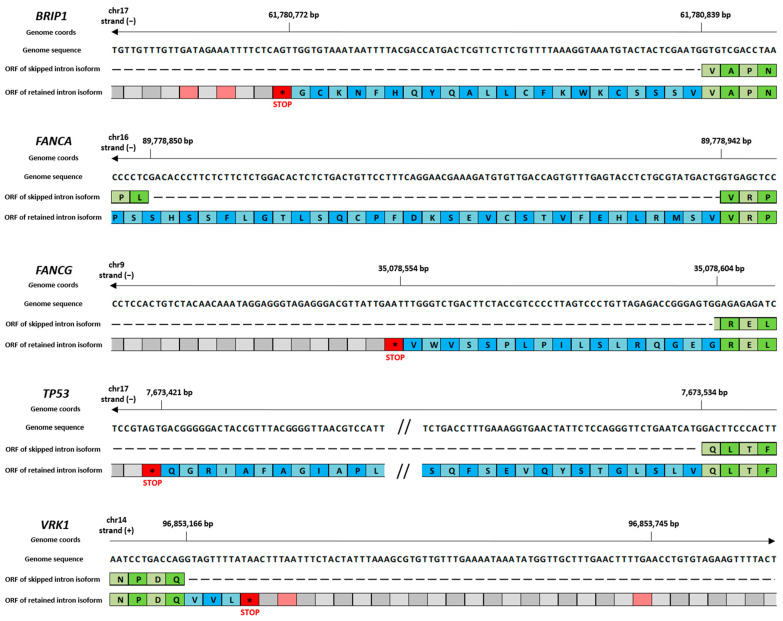
Premature stop codon identification on RI DASEs in the *BRIP1*, *FANCA*, *FANCG*, *TP53*, and *VRK1* genes. The figure was realized through a manual IGV inspection. Asterisk symbols in red boxes were used to indicate translation stop codons. Green boxes represent amino acids coded by canonical isoform, whereas the blue ones represent amino acids of the alternative isoform.

A graphical visualization of the first premature stop codon identified in the intronic region of RI DASEs was obtained with Integrative Genomics Viewer (IGV) [23] and eventually reported in Figure 4.

### 2.4. Brain-Expressed miRNAs Putatively Target DNA Repair DASE Regions

Through alternative splicing, transcripts may lose or acquire specific RNA regions containing miRNA target sites, thereby becoming either unresponsive to or subject to miRNA regulation [24]. For this reason, we investigated the potential effects of the identified Cluster 1 DASEs on miRNA interactions, hypothesizing that these splicing events could either introduce or disrupt miRNA target sites, thus altering the transcript’s susceptibility to miRNA-mediated regulation. RNAhybrid tool (V. 2.1.2) [25] was used to predict the most favorable hybridizations between our set of 15 DASEs and a list of 630 human miRNA sequences currently deposited at MirGeneDB 2.1 [26].

RNAhybrid initially predicted 5934 different miRNA–target hybridizations between the Cluster 1 DASEs and 619 distinct human miRNA sequences (Table S3).

After applying a minimum free energy (mfe) threshold (mfe ≤ −30), we retained 426 predicted alignments involving the 11 genes and 142 different miRNAs.

These DASE/miRNA predicted alignments were further classified based on seed and post-seed pairing characteristics. We applied seed annotation and excluded hybridizations not annotated with any of the predefined classes. As a result, we retained 107 DASE–miRNA hybridizations, classified as follows: 57 “Compensatory”, 47 “Canonical”, and 4 “Strong”. These hybridizations involved 8 genes and 46 unique miRNAs (Table S4).

Next, we removed 24 miRNAs that were not reported to be expressed in the brain. The remaining 22 miRNAs were analyzed for their expression levels in brain tissue. After filtering for those with expression levels above the 85th percentile, we selected nine miRNAs (hsa-miR-1301-3p, hsa-miR-423-5p, hsa-miR-127-3p, hsa-miR-766-3p, hsa-miR-210-3p, hsa-miR-874-3p, hsa-miR-877-5p, hsa-miR-887-3p, hsa-miR-671-5p). These nine miRNAs were found to hybridize with five genes: *BRCA1*, *FANCG*, *BRIP1*, *XRCC3*, and *CHEK2*, as summarized in Table 4.

### 2.5. Sulforaphane Regulates RBPs That Interact Around DASE Regions

**Figure 5:**
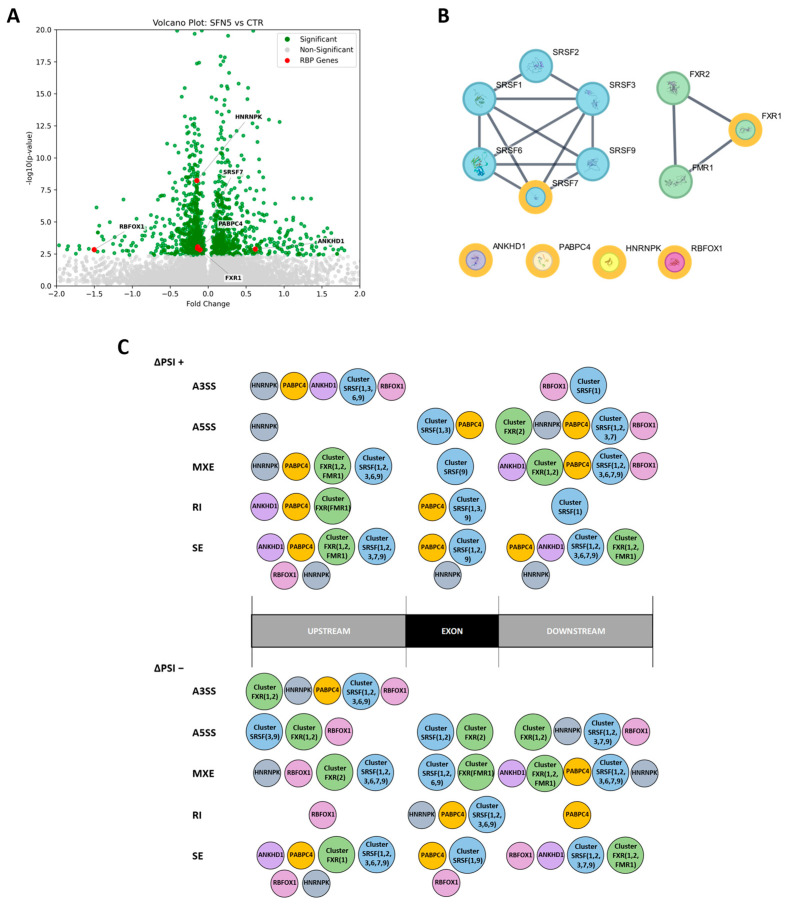
Impact of SFN on RBP genes and their binding around DASE regions. (**A**) Volcano plot of differential gene expression analysis in the SFN5 vs. CTR comparison, highlighting in red the six RBP DEGs. (**B**) Physical STRING network of RBPs: nodes with outer yellow ring are DEG-RBPs. Light-blue and green nodes represent RBPs that are not DEGs but are directly connected to DEG-RBPs by edges representing physical connections (either experimentally proved or annotated in the database). Cluster 1, represented with light-blue nodes, comprises SRSF1, SRSF2, SRSF3, SRSF6, SRSF7, and SRSF9. Cluster 2, represented by green nodes, is made up of FMR1, FXR1, and FXR2. Remaining unconnected DEG-RBPs included ANKHD1, HNRNPK, PABPC4, and RBFOX1. (**C**) Schematic representation of rMAPS2 results, illustrating motif enrichment of RBP DEGs and interactors at the upstream, exon, or downstream regions of DASEs.

Splicing factors are RBPs that interact with pre-mRNA molecules to mediate splicing processes. Moreover, it is known that specific splicing factors and other RBPs may bind around the alternative exon region to choose whether the exon region is skipped or retained, affecting the splicing pattern of each gene regulated [27]. In this context, we investigated whether SFN5 regulates RBP genes capable of interacting around the DASE regions we obtained. For this purpose, we firstly ran differential gene expression analysis to detect differentially expressed genes (DEGs) in an SFN5 vs. CTR comparison (Table S5), focusing our attention on RBP genes (listed from http://rmaps.cecsresearch.org/Help/RNABindingProtein, accessed on 9 May 2025; [28,29]). We found that SFN5 downregulated five RBP genes, i.e., *FXR1*, *HNRNPK*, *PABPC4*, *RBFOX1*, and *SRSF7*, and upregulated the *ANKHD1* gene (Figure 5A). Then, to assess the significant binding of these RBP DEGs around DASE regions, we took advantage of the rMAPS2 computational tool [30] that performs motif enrichment analysis around DASE regions. In addition to RBP DEGs, we also included in the analysis their physical interactors, since it is known that often, splicing factors operate in clusters to exert their functions [31]. To achieve this aim, we identified direct interactors by using the physical PPI STRING database. We obtained a total of 13 RBPs divided into two main clusters, “Cluster SRSF” (including SRSF1, SRSF2, SRSF3, SRSF6, SRSF7, SRSF9) and “Cluster FXR” (including FXR1, FXR2, FMR1), and then the other individual RBP DEGs (Figure 5B).

Ultimately, we ran the rMAPS2 tool and summarized its results displaying the RBPs and where they significantly bind to DASE regions (Table S6). For simplicity, we reported the exon region and the upstream and downstream regions for each splicing class and the ΔPSI values/directions. The results showed that RBP DEGs and their interactors bind around DASE regions in distinct patterns, varying according to splicing event class and ΔPSI direction. Moreover, the composition of RBP clusters associated with these interactions also differed across splicing configurations (Figure 5C). However, a common feature seemed to be the “bridge” configuration (i.e., both upstream and downstream region binding), especially for “Cluster SRSF”.

Finally, we tried to map the 13 RBPs on DNA repair DASE regions, with the aim to explore specific RBPs that may modulate AS processes on these fundamental genes. For this purpose, we took advantage of previously obtained rMAPS2 enrichment profiles, firstly scanning the 13 RBP motifs on upstream, downstream, or exon regions, and finally filtering for only those motifs that coherently bound to the regions that rMAPS2 identified (Table S7). Interestingly, “Cluster SRSF”, “Cluster FXR”, and *PABPC4* proved to overall bind our genes of interest (Table 5).

In summary, these findings report that SFN regulates some RBPs, and that they and interactors show complex binding profiles on upregulated and downregulated DASEs in the SFN5 vs. CTR comparison. Moreover, DNA repair DASEs were targeted by specific RBPs and clusters, suggesting that they may have mediated the AS observed for these genes involved in DNA repair mechanisms.

## 3. Discussion

Considering the growing body of evidence, investigating SFN’s role in AS represents a promising approach to deepen our understanding of splicing regulation and to develop targeted therapies for aberrant splicing. AS is a crucial post-transcriptional regulatory mechanism that expands transcriptomic and proteomic diversity by generating multiple mature mRNA isoforms from a single pre-mRNA transcript. This process relies on the selective inclusion or exclusion of specific exons, along with precise intron removal, resulting in transcriptomic and proteomic diversity.

Transcriptome-wide analyses showed that, on average, each protein-coding gene transcript contains 11 exons and gives rise to approximately 5.4 different mRNA isoforms [32]. The expression of AS factors is tightly regulated in a tissue-specific manner, contributing significantly to cellular differentiation and defining the molecular and functional specialization of tissues. Among all tissues, the human brain stands out as the most functionally complex, expressing a unique set of splicing factors that underlie its remarkable functional diversity [33]. However, the precise mechanisms orchestrating cell-type-specific AS regulation within such complex tissues remain largely unexplored. Additionally, AS has been demonstrated to be crucial for both the development and proper functioning of the nervous system.

In recent years, natural compounds have gained increasing attention due to their neuroprotective effects [6]. Particularly, SFN has been extensively for its ability to enhance the expression of detoxification enzymes, suppress pro-inflammatory mediators, and alleviate mitochondrial dysfunction—processes intimately involved in the pathophysiology of neurodegenerative diseases such as Alzheimer’s and Parkinson’s [6,34].

While transcriptional and epigenetic regulation by SFN has been widely documented [35,36], our study contributes novel insights by showing that SFN also modulates AS in neurons. Specifically, we evaluated the effect of SFN in modulating AS patterns, comparing RA-differentiated SH-SY5Y cells treated with 5 µM SFN (SFN5) to controls (CTR).

To systematically identify and quantify differential alternative splicing events (DASEs), we employed the rMATS turbo v4.3.0 software within a standardized analysis workflow [21]. This approach revealed that SFN treatment led to 194 significant AS events in SFN5 vs. CTR, including 92 SEs, 24 A3SSs, 41 RIs, 26 A5SSs, and 11 MXEs, underscoring the compound’s broad impact on RNA splicing dynamics.

To further elucidate the functional significance of the observed AS events, we performed a protein–protein interaction (PPI) network analysis, which revealed four distinct gene clusters. Among them, one cluster proved to be significantly relevant for downstream analyses. Indeed, it included genes involved in DNA repair mechanisms, such as X-ray Repair Cross Complementing 3 (*XRCC3*), Fanconi Anemia Complementation Group G (*FANCG*), Fanconi Anemia Complementation Group A (*FANCA*), Breast Cancer 1 (*BRCA1*), Polyhomeotic Homolog 1 (*PHC1*), BRCA1 Interacting Protein C-Terminal Helicase 1 (*BRIP1*), BRCA1-Associated RING Domain 1 (*BARD1*), Tumor Protein P53 (*TP53*), Checkpoint Kinase 2 (*CHEK2*), Vaccinia-Related Kinase 1 (*VRK1*), and DNA Polymerase Epsilon Catalytic Subunit (*POLE*). These genes are involved in genome maintenance, with a strong emphasis on DNA repair pathways. Notably, this cluster includes key components of the homologous recombination (HR) and Fanconi anemia (FA) repair systems—such as BRCA1, XRCC3, BRIP1, BARD1, FANCA, and FANCG—which act in concert to detect and resolve DNA double-strand breaks (DSBs) and inter-strand crosslinks (ICLs), two of the most cytotoxic forms of DNA damage [37,38]. The BRCA1–BARD1 complex facilitates RAD51-mediated strand invasion to promote high-fidelity repair via HR [39], while FANCA participates both within the FA core complex and independently in recombination-related mechanisms, including single-strand annealing and strand exchange [38]. The inclusion of checkpoint regulators TP53 and CHEK2 further underscores the cluster’s role in integrating DNA damage sensing with transcriptional regulation, cell cycle arrest, and apoptosis [40]. Additionally, VRK1 and PHC1 may influence repair efficiency through chromatin remodeling and modulation of p53 activity [41,42], while POLE, a high-fidelity replicative polymerase, contributes to base excision repair and the maintenance of replication accuracy [43].

Interestingly, a study by Suberbielle et al. [44] reported that BRCA1 levels are significantly reduced in the brains of Alzheimer’s disease (AD) patients and in hAPP transgenic mouse models. The authors further showed that amyloid-β oligomers downregulate BRCA1 expression in primary neurons. In vivo, selective knockdown of BRCA1 in the dentate gyrus of wild-type mice led to increased DNA double-strand breaks, neuronal atrophy, impaired synaptic plasticity, and cognitive deficits, without triggering apoptosis. These findings reveal a crucial role for BRCA1 in maintaining neuronal genome integrity and cognitive function.

Based on their biological relevance, we prioritized the analysis of these 11 genes, which collectively exhibited 15 DASEs in response to SFN treatment.

Following SFN treatment, five DNA-repair-related genes (*BRIP1*, *VRK1*, *FANCG*, *TP53*, and *FANCA*) exhibited differential splicing characterized by RIs. Notably, four of these genes (*BRIP1*, *FANCG*, *TP53*, and *FANCA*) showed negative ΔPSI values, indicating a reduction in intron-retaining isoforms after SFN exposure. In contrast, *VRK1* displayed a positive ΔPSI, suggesting increased RI. These findings suggest that SFN treatment tends to reduce RI in transcripts associated with DNA repair. To explore the functional consequences of these splicing changes, RI regions were examined for the presence of premature stop codons, which could lead to truncated and non-functional proteins. Notably, four of the five genes examined exhibited splicing isoforms predicted to introduce premature stop codons (as detailed in Table 3), which may lead to truncated, non-functional proteins or trigger nonsense-mediated mRNA decay. In contrast, one gene generated an alternatively spliced isoform that preserved the open reading frame, suggesting the potential for altered—but not lost—protein function. This analysis supports a potential mechanistic link between SFN-induced splicing alterations and modulation of gene expression, particularly in the context of DNA repair capacity.

Each RNA transcript undergoing AS can potentially be regulated by miRNAs, long non-coding RNAs (lncRNAs), and RNA-binding proteins (RBPs). As is known, dysregulation of miRNA expression has been widely implicated in the pathogenesis of neurodegenerative diseases [45,46].

Accordingly, a predictive analysis was performed to identify potential miRNA targets. The miRNA analysis encompassed all 194 DASEs to identify putative miRNAs that might bind to alternatively spliced RNA sequences, thereby potentially influencing gene expression and function. AS can create or eliminate miRNA binding sites depending on the splice variants or can alter miRNA interactions by changing the mRNA’s secondary structure. These modifications can enhance or diminish miRNA binding, resulting in upregulation or downregulation of the target transcript. miRNAs are small non-coding RNAs, approximately 22–23 nucleotides in length, that regulate gene expression post-transcriptionally by targeting specific mRNA sequences. The canonical mechanisms of miRNA-mediated repression include mRNA degradation and translational inhibition, primarily through recruitment of Argonaute proteins. miRNA–target interactions are highly diverse, involving multiple mRNA regions. While 3′ untranslated regions (3′UTRs) are the predominant sites for miRNA binding, both 5′UTRs and coding sequences (CDSs) have also been shown to participate in miRNA hybridization [47,48]. Binding specificity generally depends on complementarity within the miRNA seed region (nucleotides 2–7), often requiring an adenine at the first position, as well as matching in the post-seed region (nucleotides 13–16). Strong mRNA downregulation typically results from pairing in both regions, whereas mismatches or loops in the seed region, compensating for perfect matches in the post-seed region, tend to favor translational repression over mRNA degradation [46,49].

The analysis of DASE/miRNA interactions identified nine brain-enriched miRNAs that potentially target alternatively spliced sequences of genes involved in DNA repair. In the SFN5 vs. CTR comparison, twelve DASEs were identified, with seven exhibiting a negative ΔPSI and five showing a positive ΔPSI, indicating two A3SS, one A5SS, one MXE, three RI, and five SE events in response to SFN treatment, as detailed in Table 4. This distribution reflects the diverse splicing alterations triggered by SFN treatment. Interestingly, all three RI events exhibited a negative ΔPSI, indicating a decreased retention of these introns in the SFN5 compared to CTRL, which suggests more efficient splicing and the generation of more mature, potentially functional transcripts. This improved intron removal may be influenced by miRNAs associated with splicing regulation, including hsa-miR-127-3p, hsa-miR-766-3p, and hsa-miR-423-5p. Furthermore, hsa-miR-127-3p and hsa-miR-766-3p act together on the *BRIP* gene. Interestingly, hsa-miR-127-3p has been reported to be downregulated in the cerebrospinal fluid of frontotemporal dementia patients compared to healthy subjects [50]. Similarly, hsa-miR-766-3p has been reported to be a promising biomarker for brain aging, suggesting potential involvement in neuroinflammation and transcriptomic dysregulation [51]. In addition, hsa-miR-423-5p has been found to be significantly downregulated in Parkinson’s disease-affected brain regions, including the substantia nigra and putamen, suggesting a potential but underexplored role in dopaminergic neuron regulation and RNA processing [52,53].

To address the molecular mechanisms by which SFN produces the splicing pattern observed, we wondered whether it would regulate RBP splicing factors, and which regions they would eventually bind around DASEs.

Gene expression analysis revealed that six RNA-binding protein (RBP) genes were differentially expressed in the SFN5 vs. CTR comparison, suggesting that altered expression of these RBPs may contribute to the 194 DASEs identified, potentially driving the observed changes in AS. Specifically, five RBP genes were downregulated (*FXR1*, *HNRNPK*, *PABPC4*, *RBFOX1*, *SRSF7*) and one was upregulated (*ANKHD1*). In addition, we also considered direct and strong RBP interactors via the STRING database, obtaining two main clusters: “Cluster SRSF” (including SRSF1, SRSF2, SRSF3, SRSF6, SRSF7, SRSF9) and “Cluster FXR” (including FXR1, FXR2, FMR1). SRSF (Serine/Arginine-Rich Splicing Factor) proteins are key splicing factors, canonically known to bind to exon and intron splicing enhancers, working as splicing activators. Conversely, the HNRNP (heterogeneous nuclear ribonucleoprotein) family usually binds to exon and intron splicing silencers, working as splicing inhibitors [9]. The Fox-1 family of RNA-binding proteins, including RBFOX1, plays a critical role in the regulation of AS during neural development, with members often exhibiting partially antagonistic or context-dependent regulatory functions [54]. FMR1 (Fragile X Messenger Ribonucleoprotein 1) and its interactors, FXR1 and FXR2, are pleiotropic polyribosome-associated RBPs that play a central role in neuronal development through the regulation of alternative splicing, mRNA stability, transport, and translation [55,56,57]. Finally, PABPC4 and ANKHD1 proteins are RBPs that are not directly involved in splicing regulation but affect post-transcriptional regulation, for example, contributing to mRNA stability [58,59].

Next, we mapped the motif occurrences of the 13 RBPs on an alternative exon region and around it in the upstream and downstream regions, with the aim to identify RBP binding among ASE classes and ΔPSI directions. These RBPs significantly enriched the DASE regions, and usually more than one, especially RBPs within clusters. Interestingly, cluster composition varies among regions, ASE classes, and ΔPSI directions, suggesting the importance of specific RBPs in the splicing function, as well as the configuration of their binding along DASE regions. Overall, the binding configuration resulting from our analyses is complex to encode, even if the so-called “bridge” configuration is often adopted. It is noteworthy that RBFOX1 seems to preferentially bind to upstream regions of DASEs downregulated by SFN, suggesting that it may mediate many silenced splicing events. However, we did not find RBFOX1 enrichment among the DASE regions of the 11 genes belonging to the DNA repair process. Indeed, by mapping the binding sites of RBP DEGs and clusters on DNA repair DASE regions coherently to the rMAPS2 results, we mainly obtained SRSF and FXR clusters that bound with different compositions and configurations. The SRSF cluster bound in a “bridge” configuration (into upstream and downstream regions) for A3SS, A5SS, MXE, and SE events, whereas for RI events, it bound the exon region (i.e., the intron-retained sequence). In contrast, the FXR cluster bound into the downstream region of the *FANCG* A5SS, the SEs, and the *BRCA1* MXE DASEs. Moreover, PABPC4 specifically bound the *VRKI* RI DASE at the exon region. Finally, the RBPs FXR1, FXR2, FMR1, and PABPC4 are known to regulate not only splicing but also mRNA stability, transport, and translation processes.

These results suggest that specific RBPs modulated by SFN may mediate the splicing pattern of some crucial genes involved in DNA repair mechanisms, contributing to SFN’s physiological function and making it a potential candidate in preventing pathological disorders where DNA damage is critical, such as tumors and neurodegenerative diseases.

### Strengths and Limitations

This study aimed to verify the impact of SFN on the splicing process, highlighting the main biological processes affected by its splicing role. Thanks to this approach, we identified among the DASEs 11 genes involved in DNA repair mechanisms; however we cannot exclude other pathways regulated by SFN, as evidenced by the other 183 DASEs, deserving dedicated follow-up studies. Then, we conducted diverse predictive analyses with the aim to understand the origin and the consequences of AS patterns produced by SFN5 treatment, referring to premature stop codon analysis, DASE/miRNA hybridization, and DASE/RBP motif occurrence analysis. Despite the stringent parameters used to limit the background noise, these preliminary results should be experimentally validated to confirm their conclusions. Indeed, especially for miRNA and RBP motifs, the simple sequence match on pre-mRNA is reductive, since the effective interaction is also due to the presence of secondary structures that allow site access. Moreover, RBPs are not all splicing factors; thus the interaction we found may not be directly related to splicing processes but rather they may post-transcriptionally regulate bound mRNAs, such as transport, stability, and protein translation. To biologically support our conclusions, Western blot validation of key isoforms, and their knockdown/overexpression manipulation followed by DNA repair and genomic stability assays, should be performed; moreover, RNA immunoprecipitation, reporter assays, and perturbation of RBP or miRNA gene levels could clarify about DASE/miRNA and DASE/RBP binding. Nevertheless, our analyses were performed following a standardized approach and widely accepted tools like rMATS for event-based alternative splicing analysis [22,60,61] and rMAPS2 for RBP binding site analysis [62,63,64]. Identifying specific miRNAs and RBPs that putatively interact with specific genes of the DNA repair biological process, this study allows for future research on the novel splicing function of SFN. Finally, further studies are needed to extend these findings to in vivo models in order to assess SFN’s impact on splicing, DNA repair, and neuronal function in physiological/pathological contexts.

## 4. Materials and Methods

### 4.1. Cell Culture and Treatment

The SH-SY5Y human neuroblastoma cell line was obtained from the American Type Culture Collection (ATCC) (Manassas, VA, USA). Cells were maintained in DMEM/F-12 Ham (Sigma-Aldrich, Saint Louis, MO, USA) containing 10% Fetal Bovine Serum (FBS) (Sigma-Aldrich, Saint Louis, MO, USA), 1% L-glutamine (Sigma-Aldrich, Saint Louis, MO, USA), and 1% penicillin/streptomycin (Sigma-Aldrich, Saint Louis, MO, USA). Cells were incubated at 37 °C in a humidified atmosphere containing 5% CO_2_.

To induce their differentiation, SH-SY5Y cells were incubated for 5 days with 10 µM of RA. The SFN, produced through the bioactivation of glucoraphanin and myrosinase as described in [6], was diluted in 1× phosphate-buffered saline (PBS) (Sigma-Aldrich, Saint Louis, MO, USA). Then, differentiated cells exposed to 5 µM SFN (SFN5) and corresponding control (CTRL) samples were analyzed in our study. This concentration was selected based on our previous experiments since it proved to be both non-toxic and biologically active to regulate gene expression and induce protective responses [6].

### 4.2. RNA Extraction and cDNA Library Construction

SH-SY5Y cells were maintained and treated in 6-well plates (ThermoFisher Scientific, Rochester, NY, USA) at a seeding density of 1.5 × 10^6^ cells/well in maintenance medium. Next, cells were enzymatically dissociated using 0.25% trypsin-EDTA solution (#T4049, Sigma-Aldrich, Saint Louis, MO, USA), followed by centrifugation at 300× *g* for 5 min to collect cell pellets. Subsequently, total RNA was isolated from the cell pellet using the Maxwell^®^ RSC simplyRNA Cells Kit (#AS1390, Promega, Madison, WI, USA) on the Maxwell^®^ RSC automated extraction system, following the manufacturer’s protocol. Subsequently, library preparation was performed using 100 ng of total RNA from two biological replicates with the TruSeq^®^ RNA Exome kit (#20020189, #20020492, #20020183, #20020490; Illumina, San Diego, CA, USA), in accordance with the manufacturer’s protocol. Library, quality, and fragment distribution were assessed using the Agilent TapeStation 4150 system (Agilent, Santa Clara, CA, USA) with D1000 ScreenTape (#5067-5582 and #5067-5583). Libraries were then denatured using 0.2 N sodium hydroxide (NaOH) and diluted to a final concentration of 1.42 pM. Sequencing was carried out on the NextSeq™ 550Dx (Illumina, San Diego, CA, USA) using the NextSeq 500/550 Mid Output Reagent Kit v2.5 (150 cycles) (Illumina, San Diego, CA, USA) in paired-end mode.

### 4.3. Transcriptomic Analysis

The quality of the resulting raw paired-end reads was assessed using FastQC (version 0.11.9) (available at https://qubeshub.org/resources/fastqc, accessed on 8 January 2020). Trimmomatic (version 0.40-rc1) [65] was used to perform base clipping, remove adapters, trim for low-quality bases (at 3′ and 5′), and eventually filter out contaminants and low-quality regions. After the reads were preprocessed, they were aligned against the entire human reference genome GRCh38 deposited on Ensembl release 112 (accessed on 4 July 2024) [66] with STAR (Spliced Transcripts Alignment to a Reference) RNA-seq aligner (version 2.7.10a_alpha_220207) [67]. Aligned reads were quantified using HTSeq-count (version 0.13.5) [68], which preprocesses RNA-Seq data for differential expression analysis by counting the overlap of reads with the genes annotated in the reference genome, comprehensive of both manual and evidence-based automated annotations. Count data obtained with HTSeq-count were used as the input for the DESeq2 (version 1.36.1) [69] R package (R version 4.2.0) to directly compare gene expression levels between SFN5 and the CTR and ultimately identify differentially expressed genes (DEGs) between the conditions under investigation. This R package was used to estimate both upregulated and downregulated genes whose differential expressions were computed as fold changes (log2 ratio) according to the normalized gene expression levels in each condition, using a negative binomial. Q-values were adjusted for multiple testing using the Benjamini–Hochberg method, with a significance threshold set at 0.05. Resulting DEGs were considered significant if their corresponding adjusted *p* values were ≤0.05, computed using the Benjamini–Hochberg method [70].

### 4.4. Alternative Splicing Analysis

Alternative splicing profiles were investigated with a computational tool for Replicate Multivariate Analysis of Transcript Splicing (rMATS) for the quantification and identification of alternative splicing events between two groups of RNA samples with replicates [21]. We specifically ran rMATS for the detection of differential alternative splicing events between samples belonging to two different conditions: SFN5 and CTR, represented by two replicates each. The corresponding BAM files—binary, compressed versions of SAM (Sequence Alignment Map) files representing the output of STAR alignment against the GRCh38—were used as input to rMATS, along with a GTF (Gene Transfer Format) file describing the structure and annotation of genes and other genomic features in the human reference genome. Tool-specific flags were used to indicate the use of paired-end reads (-t paired) and a mean read length of 75 base pairs (--readLength 75). Moreover, we set 2 as the minimum number of nucleotides that must be mapped to each end of a given splice junction (--anchorLength 2) and enabled this for new, unannotated (denovo) splicing variants (--novelSS). rMATS analysis identified splicing events such as SE, MXE, A5SS, A3SS, and RI (Figure 1A), providing detailed information about the type and statistical significance of splicing changes for each event type. Specifically, the tool allowed for the quantification of PSI (Percent Spliced In) for each splicing event, indicating how often a specific exon was included in a particular region. This value ranges from 0, indicating the exon is never included, to 1, indicating the exon is always included. It is calculated based on the number of reads that unequivocally support exon inclusion versus those that unequivocally support exon exclusion. Once the PSI values were computed, rMATS than compared the PSI values cross the two experimental groups under investigation to identify alternative splicing events where the PSI values were significantly different across SFN and CTR conditions.

Finally, we filtered the resulting list of DASEs for statistical significance by retaining only those with a false discovery rate (FDR) ≤ 0.05, and further refined the selection to include events with a change in Percent Spliced In (ΔPSI) of ≥0.1 or ≤−0.1. Since low coverage splicing junctions are commonly found in RNA-seq data and frequently lead to low-confidence PSI levels, we removed low coverage events from rMATS output by setting a threshold of mean coverage > 5.

### 4.5. Protein–Protein Interaction Network from DASE Genes

The STRING (version 12.0) database (http://string-db.org/, accessed 2 July 2025) was utilized to obtain protein–protein interaction (PPI) data since it is a comprehensive database that integrates both known and predicted PPIs, including physical interactions and functional associations. It collects data from a variety of sources, such as automated text mining of the scientific literature, interaction experiment databases, co-expression data, and conserved genomic contexts, as well as transferring interaction data between species using hierarchical orthology [71]. Specifically, we investigated whether the genes affected by the resulting DASEs encoded for interacting proteins particularly connected and possibly involved in a specific common biological process.

While searching the String database, the list of DASE-affected genes was given in the input, the confidence level threshold was set to 0.7 in order to achieve only high-level confidence interactions, the species was limited to “Homo sapiens”, and the following interaction sources were included: experiments, databases, co-expression, and co-occurrence. We further manually inspected the resulting PPI network to cluster or partition it into subcomponents made by highly interconnected regions. This approach allowed for the identification of clusters representing functional modules, molecular complexes, or disease-related modules made by highly connected groups of proteins taking part to the same biological process (BP) or protein complex [72].

### 4.6. Biological Process Enrichment of Gene Cluster

GO over-representation analysis (ORA) of the biological processes (BPs) of genes in the most significantly clustered module was carried out with PANTHER (V. 19.0) (accessed on 18 March 2025) [73], available online at https://pantherdb.org, with Fisher’s exact test corrected by false discovery rate (FDR).

Over-represented biological processes were further analyzed with GOATOOLS (V. 1.4.12) [74] which enabled us to summarize the list of BPs enriched by the genes belonging to the cluster. Resulting BPs were organized by GOATOOLS in groups of related GO terms whose formal relationships were described using attributes such as “part of” and were ultimately represented with a directed acyclic graph (DAG) for greater clarity and easier interpretation of the results.

### 4.7. RI Premature Stop Codon Identification

Retained intron events can result in the generation of a premature termination codon, a stop codon that precedes the normal stop codon of a transcript.

To check for possible premature termination codons inserted by these alternative splicing events, we parsed the rMATS output to obtain RI-specific coordinates. In particular, for each RI event involving the cluster of 10 genes under investigation, we extracted the genomic coordinates spanning from the end of the exon preceding the retained intron (upstream exon) to the start of the exon following the retained intron (downstream exon). These coordinates were then used as input for the getfasta function from the BEDTools suite (v2.30.0) [75] to extract the corresponding nucleotide sequences in FASTA format directly from the GRCh38 human reference genome deposited on Ensembl [66]. Moreover, RI coordinates were loaded into the Integrative Genomics Viewer (IGV) [23] to visually inspect the retained intron and determine the reading frame (frame 1, 2, or 3) of the transcript at the RI site. Once the correct reading frame was identified, we examined RI nucleotide sequences to evaluate whether the splicing event resulted in the introduction of a novel premature termination codon. To achieve this goal, we developed and executed an in-house Python (v3.9.12) script that takes both the RI fasta sequences and their associated reading frames as input to identify the presence of in-frame premature stop codons (TAG, TAA, or TGA).

### 4.8. miRNA–DASE Hybridization Prediction

The RNAhybrid computational tool (accessed on 3 March 2025) was used to predict the most favorable miRNA–DASE hybridizations based on minimum free energy (mfe) [76]. Specifically, we were interested in the investigation of the miRNAs predicted to hybridize the subset of DASEs that involve the set of genes belonging to the cluster identified with STRING analysis. RNAhybrid was run taking two sets of sequences as input: the miRNA sequences and the target DASE sequences. The first ones were obtained from the MirGeneDB 3.0 public database (http://www.mirgenedb.org/, accessed on 13 November 2024), containing 630 miRNA sequences [26]. In contrast, DASE target sequences were extracted from the GRCh38 human reference genome (Ensembl) using the getfasta function from the BEDTools suite (v2.30.0) [75]. These extractions were based on DASE coordinates provided in BED format, which were derived following rMATS guidelines for each alternative splicing event type, as detailed in Table 6.

Only hybridizations with an mfe lower than −30 kcal/mol were retained, as this threshold was proposed in previous studies to warrant high-confidence interactions [76]. The resulting miRNA-DASE predicted alignments were then categorized into three classes: canonical, strong, and compensatory [77,78]. Canonical labels were assigned to hybridizations characterized by perfect Watson–Crick base pairings (A–U and G–C) within the seed region (positions 2–7) only. Strong alignments were the ones including perfect base pairings in both seed and post-seed regions (positions 13–16). Lastly, compensatory interactions were defined by mismatches within the seed region that were offset by complementary base pairing in the post-seed region. Each predicted hybridization was ultimately annotated for the adenosine (A) presence opposite position 1 of the miRNA, which has been reported to be crucial for Argonaute binding and target recognition [77]. RNA hybrid alignments not fitting any of the three structural classes were excluded.

Lastly, miRNA-DASE predicted alignments were filtered to ensure biological consistency of the results. With this aim, we retained only hybridizations involving miRNAs highly expressed in brain tissue. Expression profiles were downloaded from miRNATissueAtlas2 [79,80] (accessed on 16 May 2024), a publicly available resource containing non-coding RNA data from multiple NGS experiments. The database was parsed in order to obtain a list of miRNAs that were annotated to be expressed in brain tissue, and ultimately filtered to obtain those with expression levels between the 85th percentile and the maximum observed expression value (high-expression range). We ultimately retained a set of miRNA–DASE hybridizations involving miRNAs that were annotated with at least one of the defined structural classes and exhibited expression levels in the brain within the high-expression range.

### 4.9. Sashimi Plots

Sashimi plots were generated with rmats2sashimiplot version 3.0.0 to visually and quantitatively represent splice junctions and exon usage based on mRNA alignments to an annotated genome, enabling comparison of alternative splicing across conditions. Specifically, this tool uses transcript annotations together with spliced read alignments of each sample, and visualizes each region of interest by displaying exon alignments as read density plots and depicting splice junction reads as arcs connecting pairs of exons, with the arc thickness proportional to the number of reads mapping on the junction [81].

The annotation file of the human reference genome GRCh38 in GFF3 (Generic Feature Format), along with spliced read alignments in BAM (Binary Alignment Map) format, were provided as input to rmats2sashimiplot. A total of four BAM files were used, corresponding to two biological replicates per group: CTR and SFN5.

For each group, the tool calculated the average inclusion level, average read depth, and average number of junction-spanning reads, enabling a quantitative comparison of alternative splicing events between the two conditions.

### 4.10. RBP Motif Occurrence Analysis

To investigate the regulatory role of RNA-binding proteins (RBPs) in the context of alternative splicing events, we performed a computational analysis focused on RBP–pre-mRNA interactions. Given that RBPs belong to a class of protein that represents key splicing regulators, we analyzed their binding potential around DASEs resulting from our analysis [82,83]. This was achieved using the well-established rMAPS2 (RNA Map Analysis and Plotting Server 2), a computational motif enrichment analysis tool for RNA-binding proteins, freely available at http://rmaps.cecsresearch.org/ (accessed on 14 May 2025), designed to predict protein–RNA interactions based on the presence of known RBP recognition motifs near alternative splicing events [30,84]. Specifically, we used rMAPS2 (accessed on 14 May 2025) to analyze differential alternative splicing data obtained from rMATS and eventually identify enriched RNA-binding protein target sites. This tool took in as input AS events from rMATS output, genome assembly type (hg38 as it represents human reference genome), and a preloaded list of 114 RBP motifs [28,29]. It calculated motif density using a sliding window of 50 nucleotides across the associated exonic regions (±50 nt) and flanking intronic regions (±250 nt). The regions within this interval were sequentially labeled from R1 to Rn, where n corresponds to the total number of regions defined for each event type. Motif scores were computed separately for the set of upregulated (positive ΔPSI), downregulated (negative ΔPSI), and background (nonregulated) exons, and were ultimately plotted in red, blue, and black solid lines, respectively. rMAPS2 performed a Wilcoxon rank sum statistical test to highlight significant differences in motif presence for each sliding window, and plotted negative log10 *p* values with red and blue dotted lines for upregulated versus background and downregulated versus background, respectively.

### 4.11. RBP Motif Analysis Output Parsing

To further refine the results from rMAPS2 analysis on RBPs and better understand their biological context, we created a PPI network of interacting RBPs. Specifically, we used STRING (accessed on 4 July 2025) to represent the physical network underneath RBPs whose recognition motifs near alternative splicing events were listed in the rMAPS2 tool.

Starting from this set of proteins, we constructed a physical subnetwork using a minimum required interaction score of 0.900 including ‘Experiments’ and ‘databases’ as the only active interaction sources. The resulting network represented the highest-confidence interactome with edges indicating physical interactions—either experimentally validated or reported in curated databases—suggesting that the connected proteins were part of the same physical complex. The nodes of the resulting PPI network were categorized into two groups: RBPs encoded by genes differentially expressed following SFN5 treatment (DEG RBPs), and RBPs encoded by genes whose expression remained unchanged (non-DEG RBPs). We removed from the network all non-DEG RBP nodes that were not directly or indirectly connected to any DEG RBP node. The remaining network was ultimately composed of two different subnetworks of interacting nodes and unconnected DEG RBPs (Figure 5B).

We analyzed rMAPS2 results to identify significant differences in motif presence, focusing specifically on RBPs included in our final PPI network. For simplicity, regions from R1 to Rn were divided into three main regions: “Upstream”, for those regions before the exonic ones; “Exon”, for the regions relative to the alternative exon; and “Downstream”, for the regions after the exonic ones. Accordingly, in Figure 5C, the region-binding of each RBP in the network was reported.

Lastly, we documented rMAPS2 results specifically for each DASE of interest (i.e., the 15 DASEs related to DNA repair genes), developing a custom Python (v3.9.12) script that examines defined regions based on exonic (±50 nt) and flanking intronic (±250 nt) sequences. It extracts the corresponding nucleotide sequences and scans them for motifs associated with each RBP in the network. For each region of every DASE, the script assesses and retains only RBP motif occurrences that align with the enrichment patterns identified by rMAPS2, according to its specific positional information and ΔPSI and ASE class, ensuring consistency and biological relevance in the final output. Finally, upstream, exon, and downstream regions were properly annotated for each RBP (Table S7).

## 5. Conclusions

Our study reports that SFN modulates the AS patterns of genes involved in DNA repair and the DNA damage response in an in vitro neuronal model. Notably, SFN appears to influence the expression of DNA repair genes by modulating the inclusion of isoforms that harbor intronic premature stop codons or are regulated by miRNAs and RBPs. These factors may collectively affect transcripts’ maturation, stability, localization, and translation. These findings uncover a novel mechanism by which SFN, widely recognized to transcriptionally modulate genes and pathways involved in antioxidant and cytoprotective processes, may contribute to neuroprotection also through the regulation of splicing events in genes critical for maintenance of DNA integrity. By promoting the fine-tuning of DNA repair gene expression, SFN may help mitigate DNA damage caused by oxidative stress, ultimately supporting neuronal integrity and function. Importantly, our results suggest that SFN may serve not only as a therapeutic candidate in pathological contexts such as neurodegenerative diseases, where oxidative stress and impaired DNA repair are common, but also as a valuable adjuvant in physiological conditions to enhance cellular resilience through its dual activity. Further studies are warranted to confirm these preliminary findings and to elucidate the precise molecular mechanisms underlying SFN’s impact on alternative splicing and DNA repair in neuronal cells. A deeper understanding of this interplay could pave the way for novel preventive or therapeutic strategies targeting neurodegenerative and age-related disorders.

## Figures and Tables

**Table 1:** List of the most enriched biological processes (BPs) by Cluster 1 and Cluster 2, ordered by Fold Enrichment values.

PANTHER GO-Slim BP	Cluster	# Genes	Fold Enrichment	Raw *p* Value	FDR
nucleotide-excision repair	1	2	>100	9.28 × 10^−5^	2.46 × 10^−2^
DNA recombination	1	3	54.16	2.03 × 10^−5^	8.63 × 10^−3^
double-strand-break repair	1	3	50.94	2.44 × 10^−5^	8.64 × 10^−3^
mRNA processing	2	3	51.88	1.89 × 10^−5^	4.01 × 10^−2^
mRNA metabolic process	2	3	37.85	4.85 × 10^−5^	3.43 × 10^−2^
DNA repair	1	4	35.73	3.43 × 10^−6^	2.42 × 10^−3^
DNA damage response	1	5	34.86	1.73 × 10^−7^	3.68 × 10^−4^
DNA metabolic process	1	4	23.41	1.82 × 10^−5^	9.66 × 10^−3^
cellular response to stress	1	5	20.08	2.64 × 10^−6^	2.80 × 10^−3^
RNA metabolic process	2	4	17.53	3.62 × 10^−5^	3.85 × 10^−2^
response to stress	1	5	10.60	5.83 × 10^−5^	1.77 × 10^−2^

**Table 2:** The table reports Cluster 1 DASEs with relative ΔPSI values and FDRs.

ASE Class	GeneID	Gene Symbol	ΔPSI SFN5 vs. CTR	FDR
A3SS	ENSG00000126215	*XRCC3*	0.357	0.0043
A3SS	ENSG00000221829	*FANCG*	−0.169	0.0350
A3SS	ENSG00000187741	*FANCA*	−0.173	0.0382
A3SS	ENSG00000177084	*POLE*	0.152	0.0330
A5SS	ENSG00000221829	*FANCG*	−0.301	0.0070
MXE	ENSG00000012048	*BRCA1*	0.111	0.0468
RI	ENSG00000136492	*BRIP1*	−0.291	0.0084
RI	ENSG00000100749	*VRK1*	0.107	0.0451
RI	ENSG00000221829	*FANCG*	−0.326	0.0028
RI	ENSG00000141510	*TP53*	−0.378	0.0037
RI	ENSG00000187741	*FANCA*	−0.194	0.0123
SE	ENSG00000183765	*CHEK2*	0.293	0.0316
SE	ENSG00000138376	*BARD1*	0.254	0.0140
SE	ENSG00000221829	*FANCG*	−0.162	0.0356
SE	ENSG00000111752	*PHC1*	−0.284	0.0399

**Table 3:** The table reports the RI class of DNA repair DASEs with the annotation of premature stop codons encountered.

Gene	RI Coordinates	ΔPSI SFN5 vs. CTR	Frame of ORF	No. of Premature Stop Codons	Position of the First Stop Codon
*BRIP1*	chr17:61776562-61780839 (−)	−0.291	Frame 1	83	67
*FANCA*	chr16:89778850-89778942 (−)	−0.194	Frame 1	No stop codons	---
*FANCG*	chr9:35077399-35078604 (−)	−0.326	Frame 3	14	51
*TP53*	chr17:7673339-7673534 (−)	−0.378	Frame 1	2	115
*VRK1*	chr14:96853166-96855223 (+)	0.107	Frame 1	50	10

**Table 4:** List of miRNA–target hybridizations predicted by RNAhybrid tool with a mfe ≤ −30 and involving miRNAs highly expressed in brain tissue.

ASE Class	Gene	miRNA	ΔPSI SFN5 vs. CTR	mfe (kcal/mol)
A3SS	*XRCC3*	hsa-miR-210-3p	0.357	−33.8
A3SS	*FANCG*	hsa-miR-423-5p	−0.169	−30.3
A5SS	*FANCG*	hsa-miR-423-5p	−0.301	−30.3
MXE	*BRCA1*	hsa-miR-1301-3p	0.111	−30.4
RI	*BRIP1*	hsa-miR-127-3p	−0.291	−31.5
RI	*BRIP1*	hsa-miR-766-3p	−0.291	−30.4
RI	*FANCG*	hsa-miR-423-5p	−0.326	−30.3
SE	*CHEK2*	hsa-miR-874-3p	0.293	−30.6
SE	*CHEK2*	hsa-miR-877-5p	0.293	−36.9
SE	*CHEK2*	hsa-miR-887-3p	0.293	−35
SE	*FANCG*	hsa-miR-423-5p	−0.162	−30.3
SE	*FANCG*	hsa-miR-671-5p	−0.162	−30.7

**Table 5:** List of RBP DEGs and clusters interacting around DNA repair DASE regions.

Class (∆PSI)	DASE Gene Name	CLUSTER and RBP DEGs		
		Upstream	Exon	Downstream
A3SS (−)	*FANCA*	---	---	---
A3SS (−)	*FANCG*	Cluster SRSF (1, 2, 3, 9)	---	---
A3SS (+)	*POLE*	Cluster SRSF (1)	---	---
A3SS (+)	*XRCC3*	Cluster SRSF (1, 3, 9)	---	---
A5SS (−)	*FANCG*	Cluster SRSF (3)	---	Cluster SRSF (1, 3, 9); Cluster FXR (2)
MXE (+)	*BRCA1*	Cluster SRSF (3)	---	Cluster SRSF (3, 6); Cluster FXR (1)
RI (−)	*BRIP1*	---	Cluster SRSF (1, 2, 3, 9)	---
RI (−)	*FANCA*	---	---	---
RI (−)	*FANCG*	---	Cluster SRSF (1, 2, 6)	---
RI (−)	*TP53*	---	Cluster SRSF (3, 6)	---
RI (+)	*VRK1*	---	Cluster SRSF (3, 9); PABPC4	---
SE (+)	*BARD1*	Cluster SRSF (1, 3)	---	Cluster SRSF (1, 2, 3, 9)
SE (+)	*CHEK2*	Cluster SRSF (2, 3)	Cluster SRSF (9)	Cluster SRSF (2, 3, 9)
SE (−)	*FANCG*	Cluster SRSF (1, 2, 3, 9)	---	Cluster SRSF (1, 2, 3); Cluster FXR (2, FMR1)
SE (−)	*PHC1*	Cluster SRSF (2, 3)	---	Cluster SRSF (3)

**Table 6:** Rules used to extract nucleotide sequences from DASE coordinates: Starting and stopping positions were determined based on the splicing event type and strand orientation, following the guidelines from the rMATS manual. For A3SS and A5SS events, overlapping regions with the short exon were excluded from the long exon sequence.

DASE Type	Strand	Start Position	End Position
SE	+ and −	Exon Start	Exon End
MXE	+	First Exon Start	First Exon End
	−	Second Exon Start	Second Exon End
RI	+ and −	Upstream Exon End	Downstream Exon Start
A3SS *	+ and −	Long Exon Start	Long Exon End
A5SS *	+ and −	Long Exon Start	Long Exon End

* Excluding overlap with short exon region.

## Data Availability

All data generated and analyzed during this study are available in the NCBI SRA database under the BioProject accession number PRJNA1291672.

## Supplementary Materials

The following supporting information can be downloaded at: https://www.mdpi.com/article/10.3390/ijms26178187/s1.

## Abbreviations

The following abbreviations are used in this manuscript:

3′UTRs	3′untranslated regions
5′UTRs	5′untranslated regions
A	adenosine
AS	alternative splicing
A3SS	alternative 3′ splice site
A5SS	alternative 5′ splice site
ATCC	American Type Culture Collection
BAM	Binary Alignment Map
BPs	biological processes
CDSs	coding sequences
CTR	control
DAG	directed acyclic graph
DASEs	differential alternative splicing events
DEGs	differentially expressed genes
DDR	DNA damage response
FDR	false discovery rate
FBS	Fetal Bovine Serum
GFF3	Generic Feature Format
GO	Gene Ontology
GTF	Gene Transfer Format
IGV	Integrative Genomics Viewer
mfe	minimum free energy
miRNAs	microRNAs
MXE	mutually exclusive exon
ORA	over-representation analysis
PPI	protein–protein interaction
PSI	Percent Spliced In
RA	retinoic acid
RBP	RNA-binding protein
RI	retained intron
rMATS	replicate Multivariate Analysis of Transcript Splicing
SAM	Sequence Alignment Map
SE	skipped exon
SFN	sulforaphane
SFN5	5 µM SFN
snRNPs	small nuclear ribonucleoproteins
SREs	splicing regulatory elements
STAR	Spliced Transcripts Alignment to a Reference
