# Supplementary material for: Computational Splicing Analysis of Transcriptomic Data Reveals Sulforaphane Modulation of Alternative mRNA Splicing of DNA Repair Genes in Differentiated SH-SY5Y Neurons
Source: Int J Mol Sci. 2025 Aug 23;26(17):8187. doi: 10.3390/ijms26178187 (PMC12428557; doi:10.3390/ijms26178187)
Supplement: Supplementary file 1 [file ijms-26-08187-s001.zip › Figure S2.pdf]

chr14_103711033_103711180_-@chr14_103711033_103711245_-@chr14_103711466_103711567_-

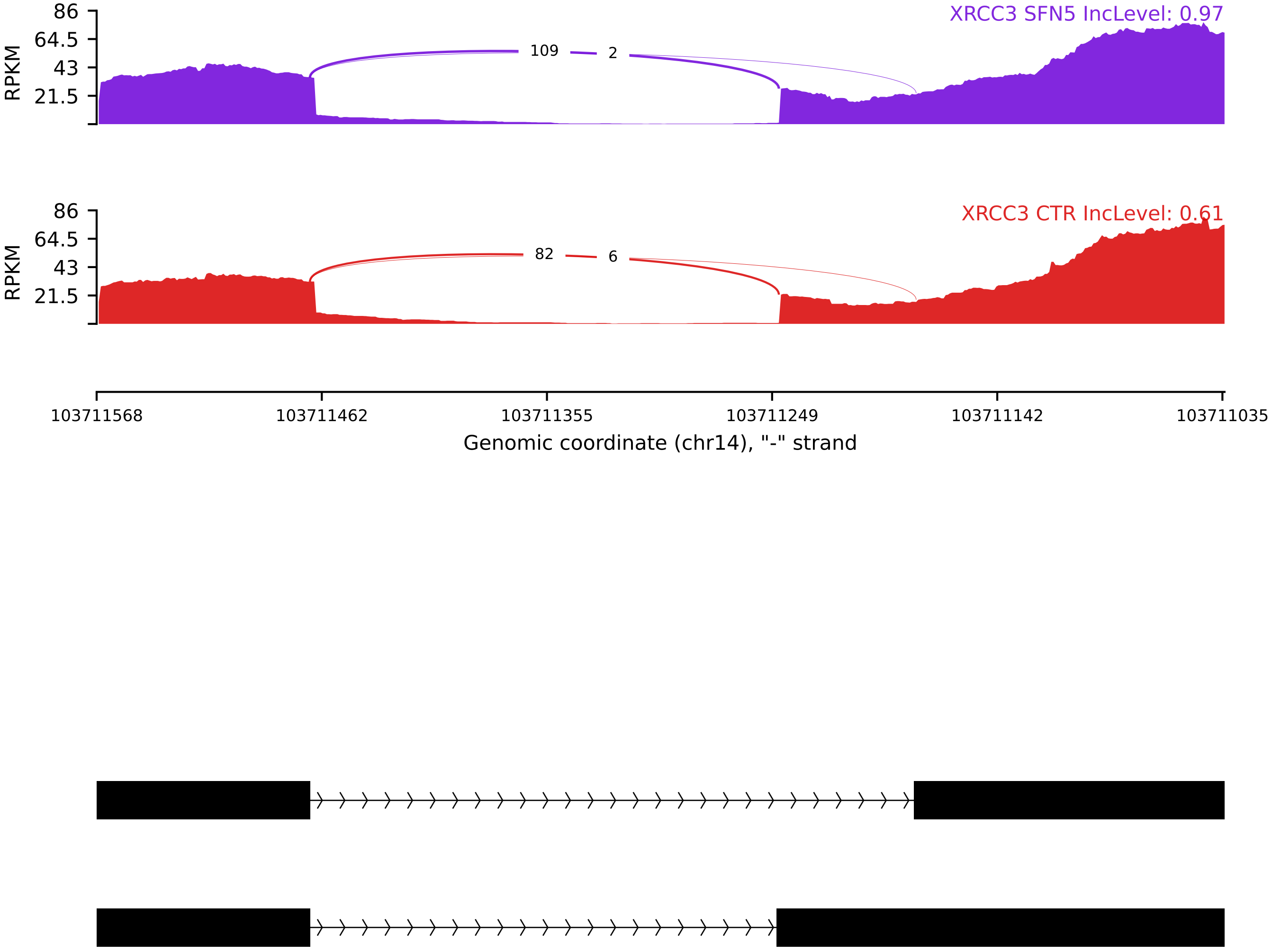

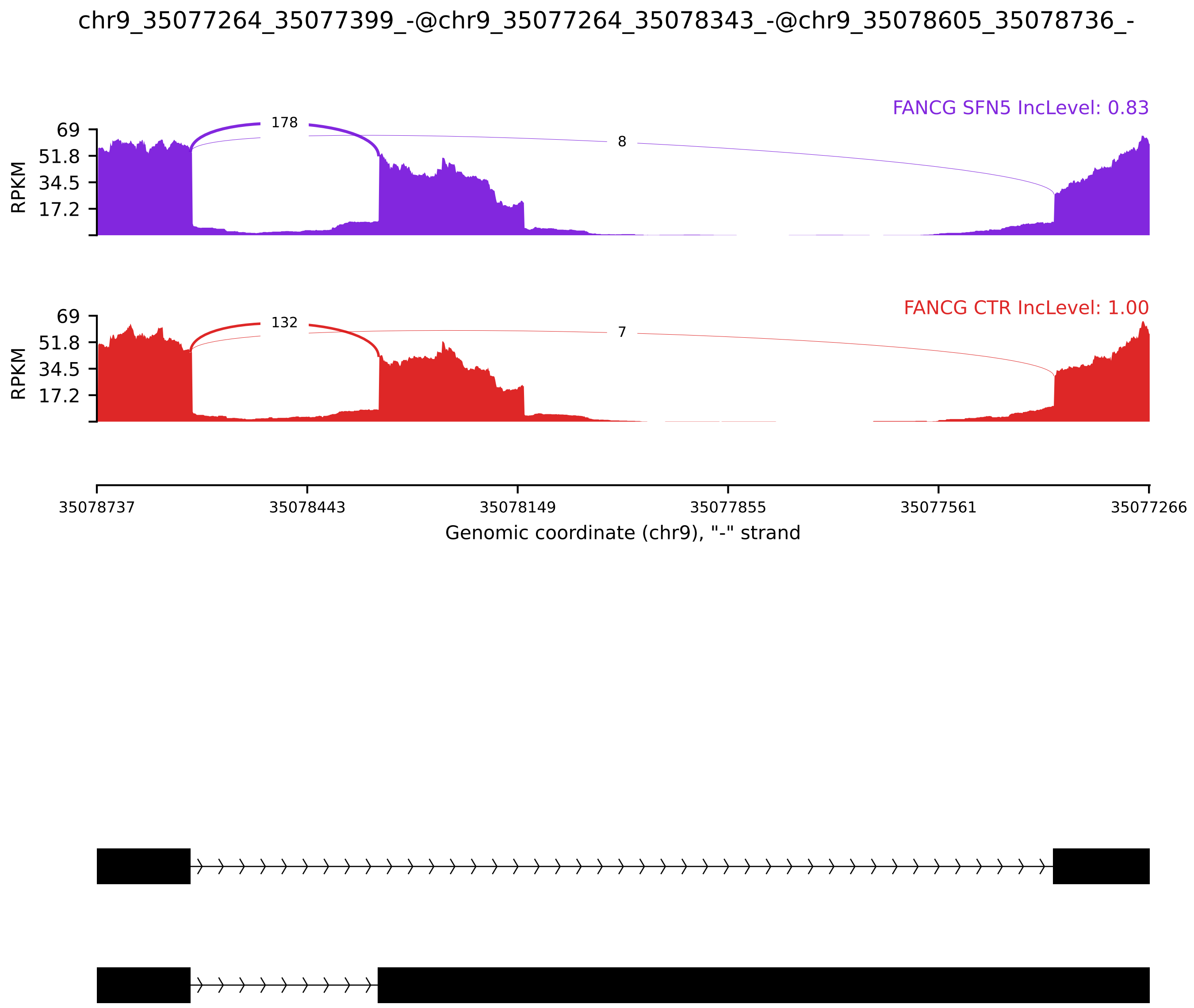
chr9_35077264_35077399_-@chr9_35077264_35078343_-@chr9_35078605_35078736_-
FANCG SFN5 IncLevel: 0.83
69
51.8
34.5
17.2
RPKM
178
8
FANCG CTR IncLevel: 1.00
69
51.8
34.5
17.2
RPKM
132
7
35078737
35078443
35078149
35077855
35077561
35077266
Genomic coordinate (chr9), "-" strand

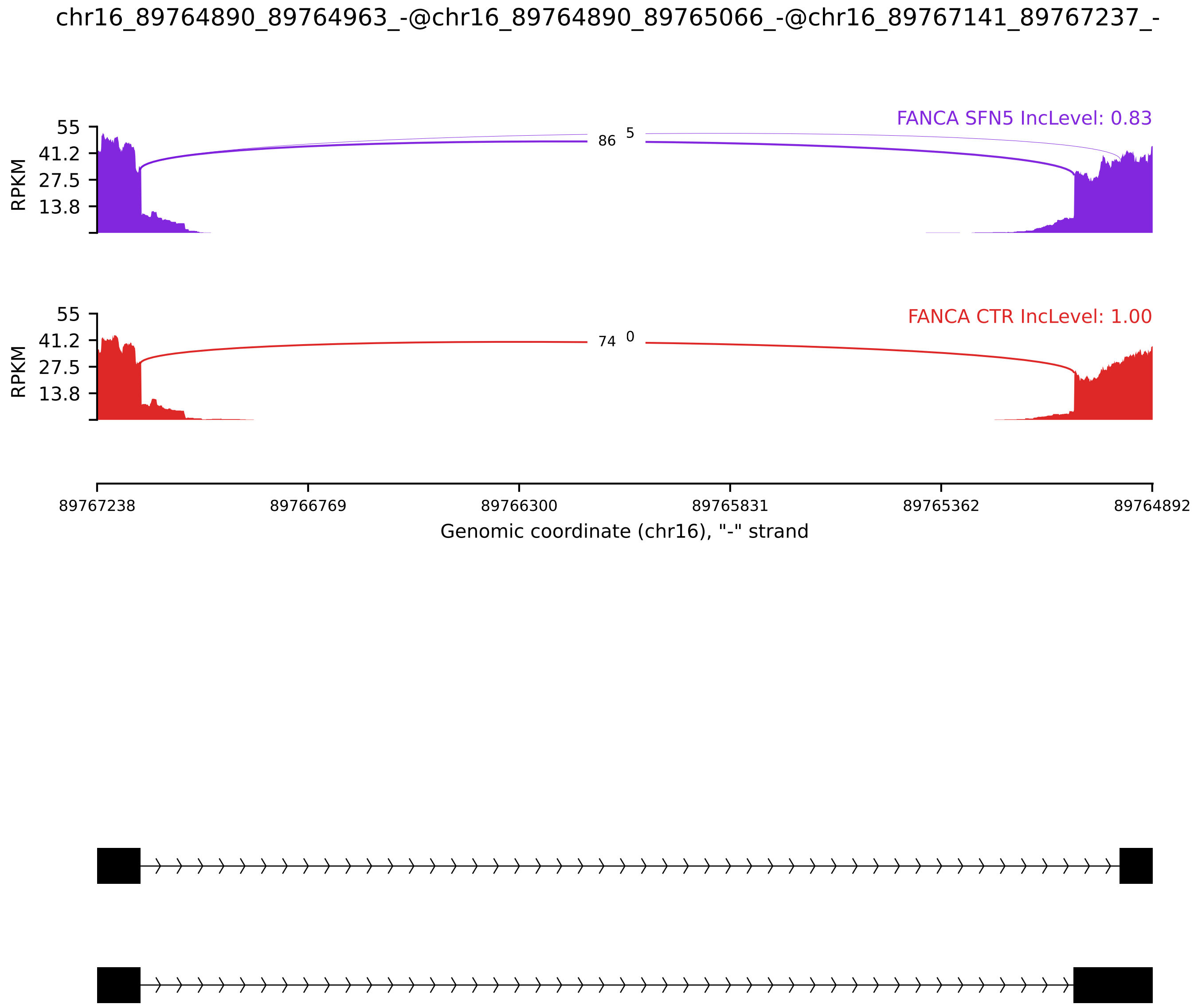
chr16_89764890_89764963_-@chr16_89764890_89765066_-@chr16_89767141_89767237_-
FANCA SFN5 IncLevel: 0.83
RPKM
55
41.2
27.5
13.8
86
5
FANCA CTR IncLevel: 1.00
RPKM
55
41.2
27.5
13.8
74
0
89767238
89766769
89766300
89765831
89765362
89764892
Genomic coordinate (chr16), "-" strand

chr12_132664370_132664415_-@chr12_132664370_132664462_-@chr12_132665302_132665450_-

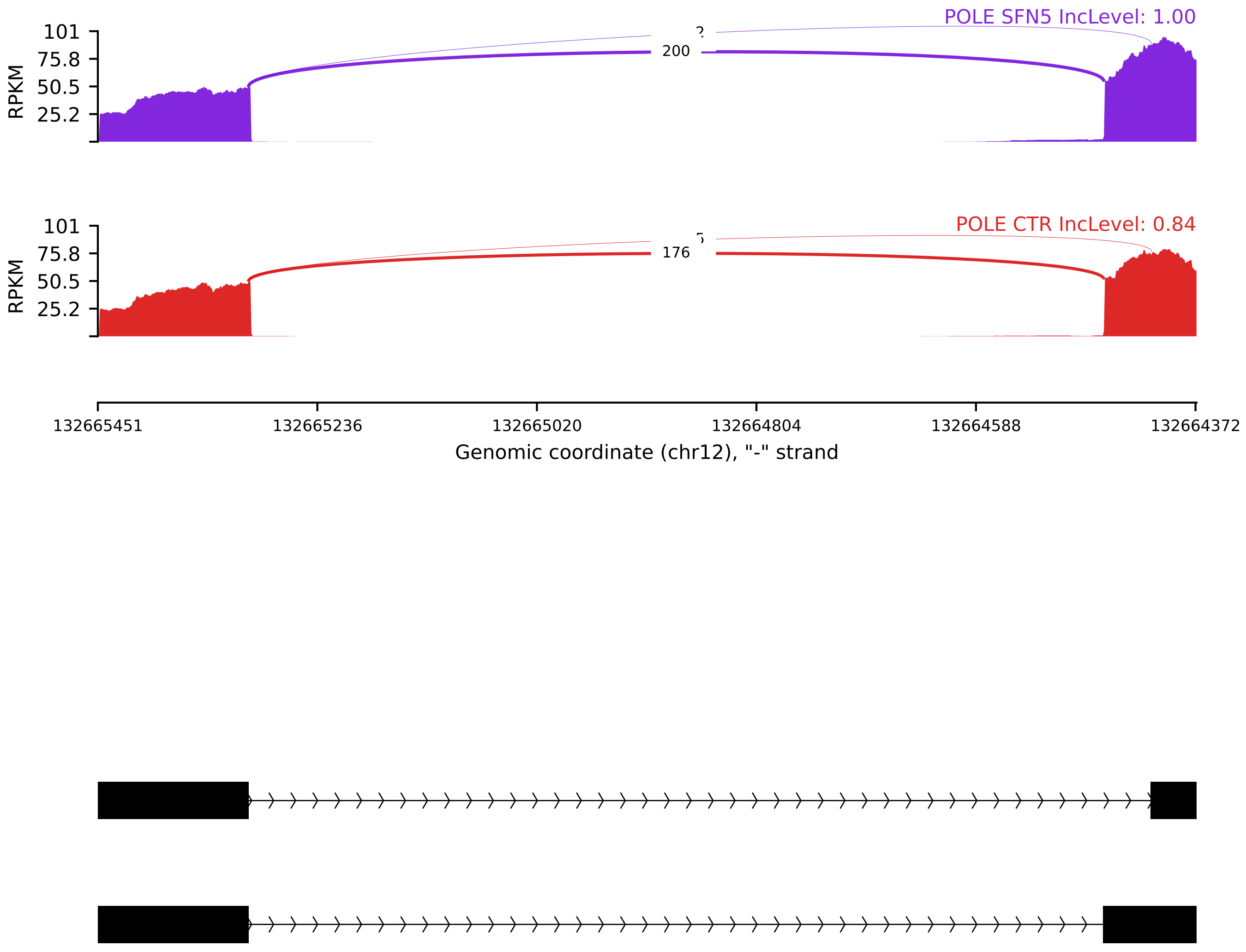

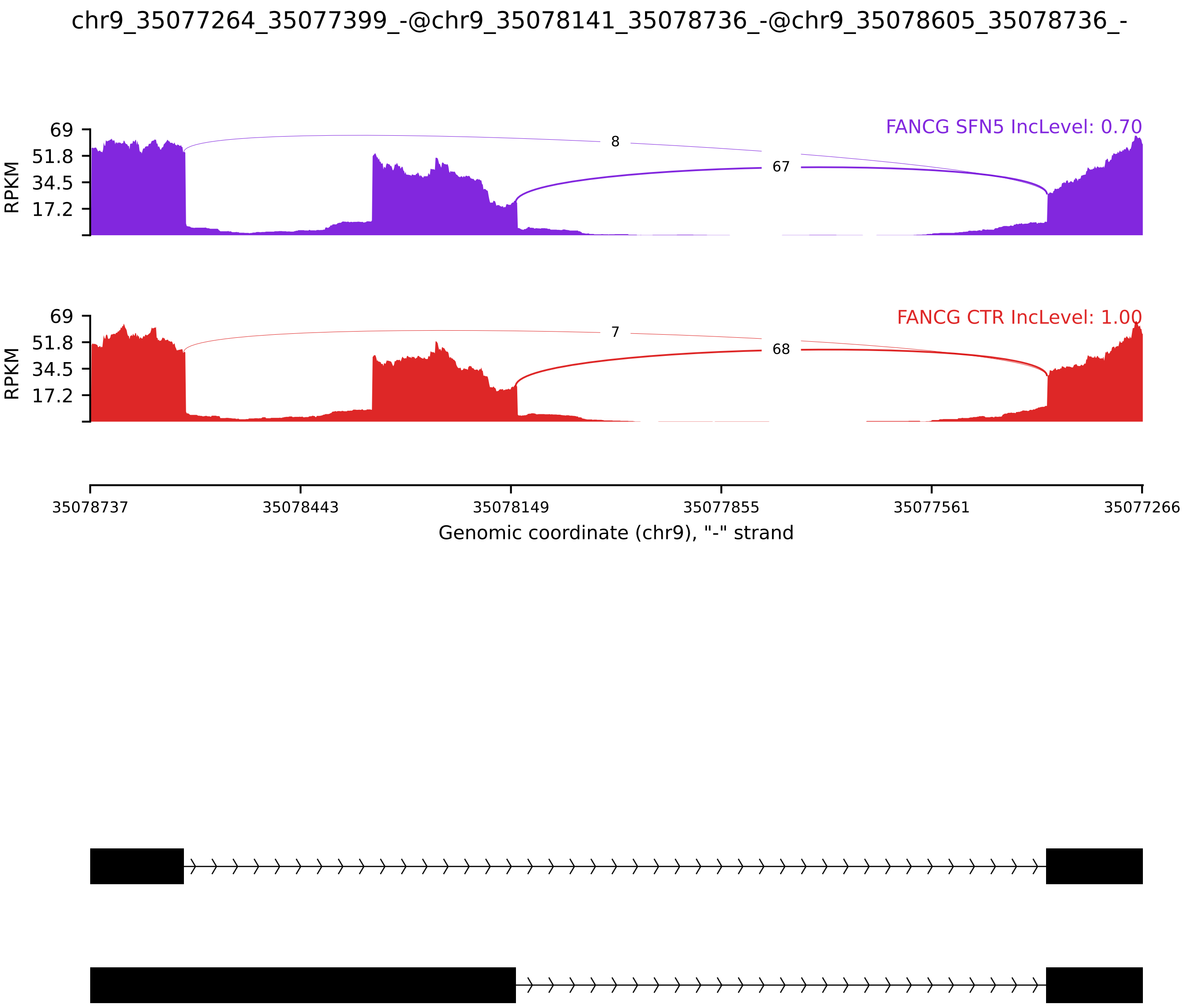
chr9_35077264_35077399_-@chr9_35078141_35078736_-@chr9_35078605_35078736_-
FANCG SFN5 IncLevel: 0.70
8
67
FANCG CTR IncLevel: 1.00
7
68
RPKM
69
51.8
34.5
17.2
35078737
35078443
35078149
35077855
35077561
35077266
Genomic coordinate (chr9), "-" strand

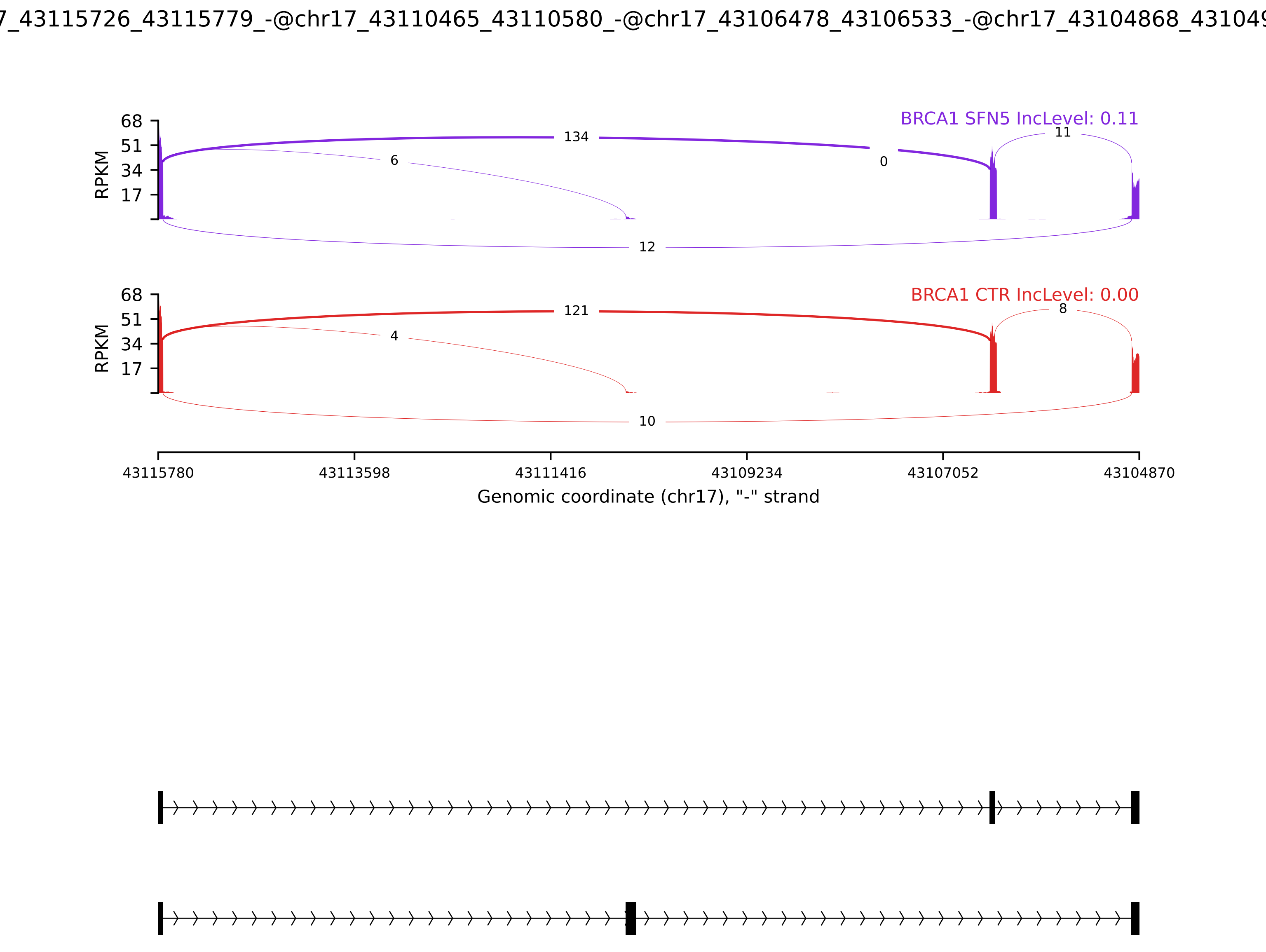
7_43115726_43115779_-@chr17_43110465_43110580_-@chr17_43106478_43106533_-@chr17_43104868_43104[illegible]
BRCA1 SFN5 IncLevel: 0.11
RPKM
68
51
34
17
134
6
0
11
12
BRCA1 CTR IncLevel: 0.00
RPKM
68
51
34
17
121
4
8
10
43115780
43113598
43111416
43109234
43107052
43104870
Genomic coordinate (chr17), "-" strand

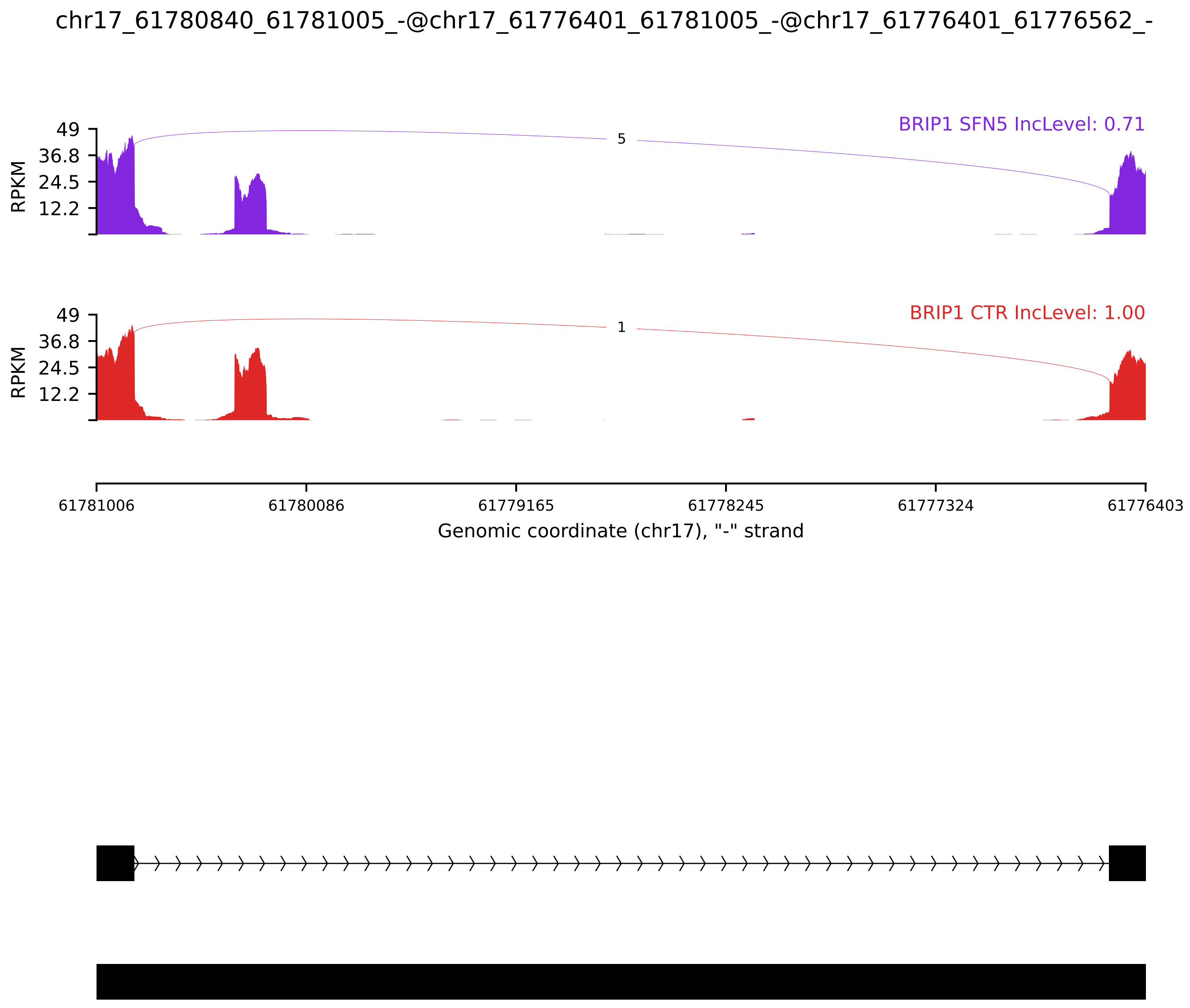

chr17_61780840_61781005_-@chr17_61776401_61781005_-@chr17_61776401_61776562_-
BRIP1 SFN5 IncLevel: 0.71
BRIP1 CTR IncLevel: 1.00
RPKM
49
36.8
24.5
12.2
5
1
61781006
61780086
61779165
61778245
61777324
61776403
Genomic coordinate (chr17), "-" strand

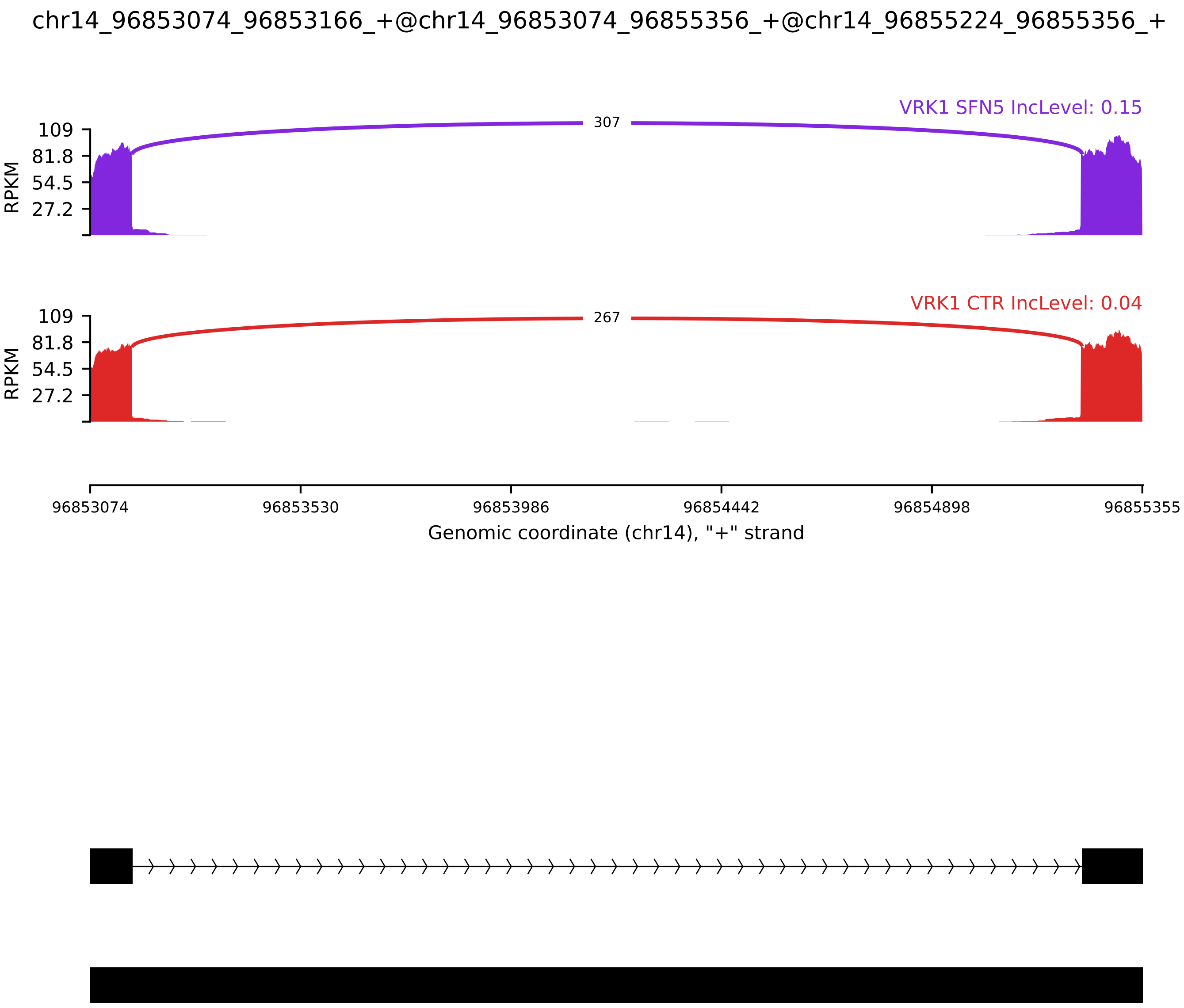
chr14_96853074_96853166_+@chr14_96853074_96855356_+@chr14_96855224_96855356_+
VRK1 SFN5 IncLevel: 0.15
307
109
81.8
54.5
27.2
RPKM
VRK1 CTR IncLevel: 0.04
267
109
81.8
54.5
27.2
RPKM
96853074
96853530
96853986
96854442
96854898
96855355
Genomic coordinate (chr14), "+" strand

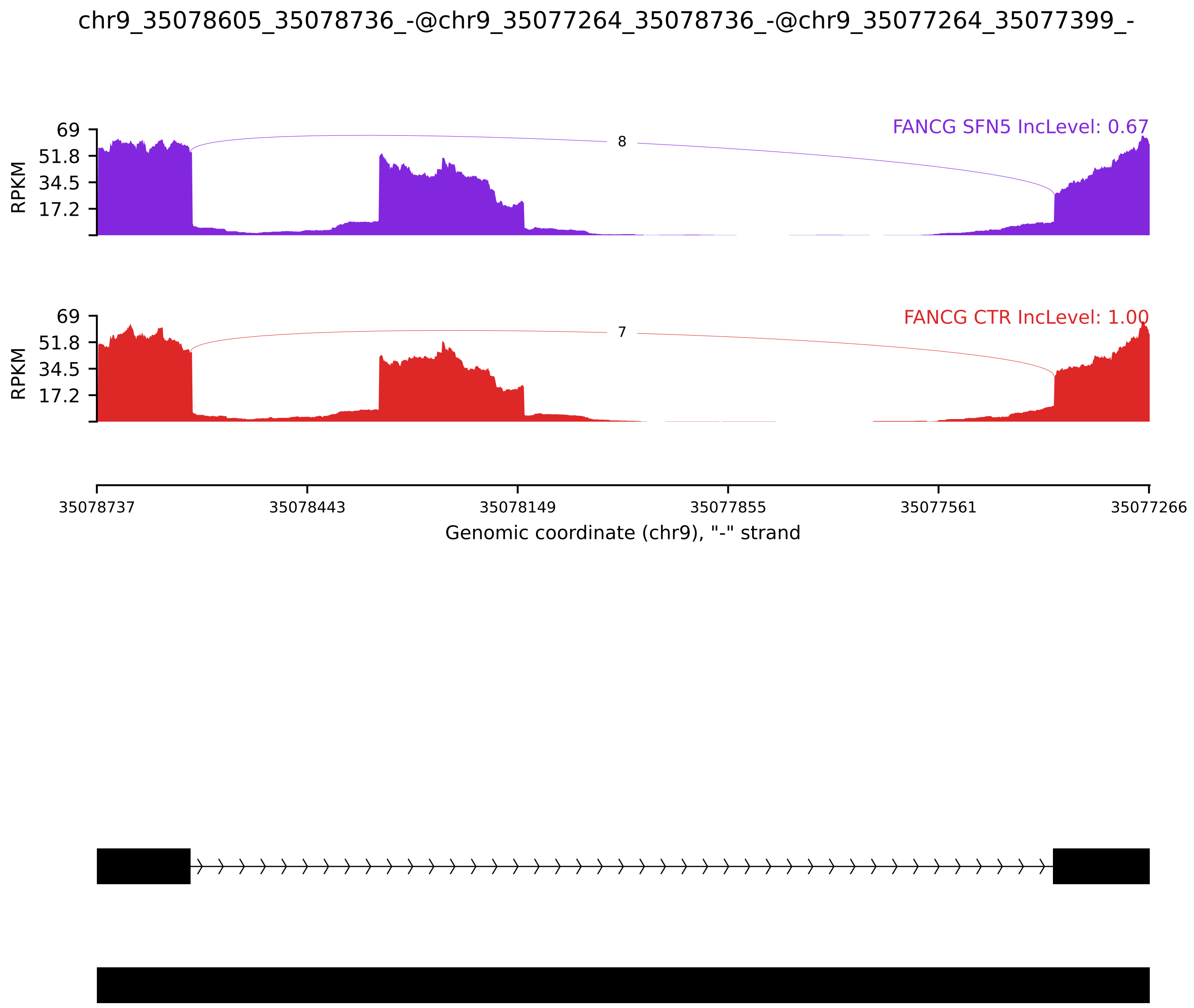
chr9_35078605_35078736_-@chr9_35077264_35078736_-@chr9_35077264_35077399_-
FANCG SFN5 IncLevel: 0.67
FANCG CTR IncLevel: 1.00
RPKM
69
51.8
34.5
17.2
8
7
35078737
35078443
35078149
35077855
35077561
35077266
Genomic coordinate (chr9), "-" strand

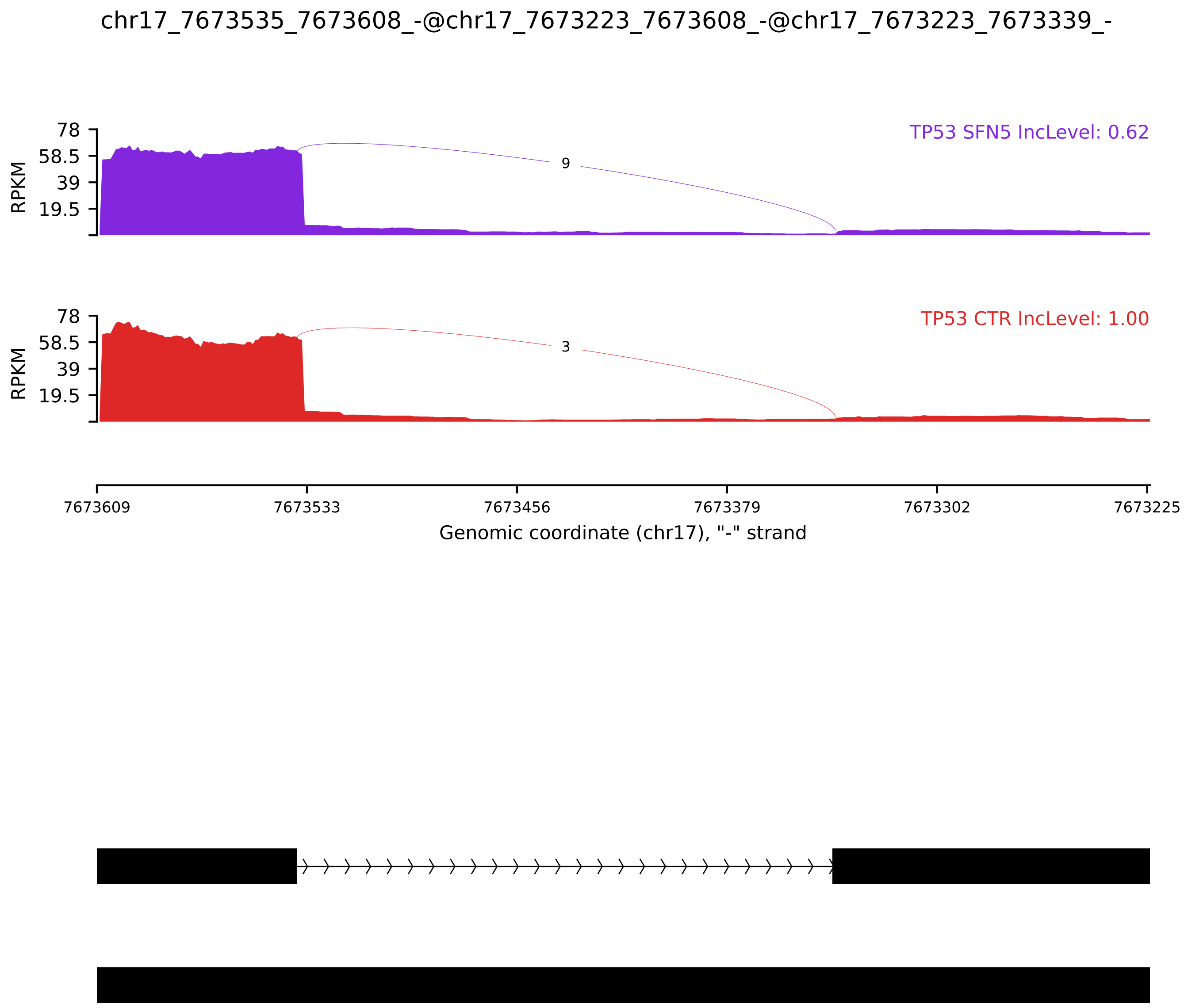

chr17_7673535_7673608_-@chr17_7673223_7673608_-@chr17_7673223_7673339_-
TP53 SFN5 IncLevel: 0.62
TP53 CTR IncLevel: 1.00
RPKM
78
58.5
39
19.5
9
3
7673609
7673533
7673456
7673379
7673302
7673225
Genomic coordinate (chr17), "-" strand

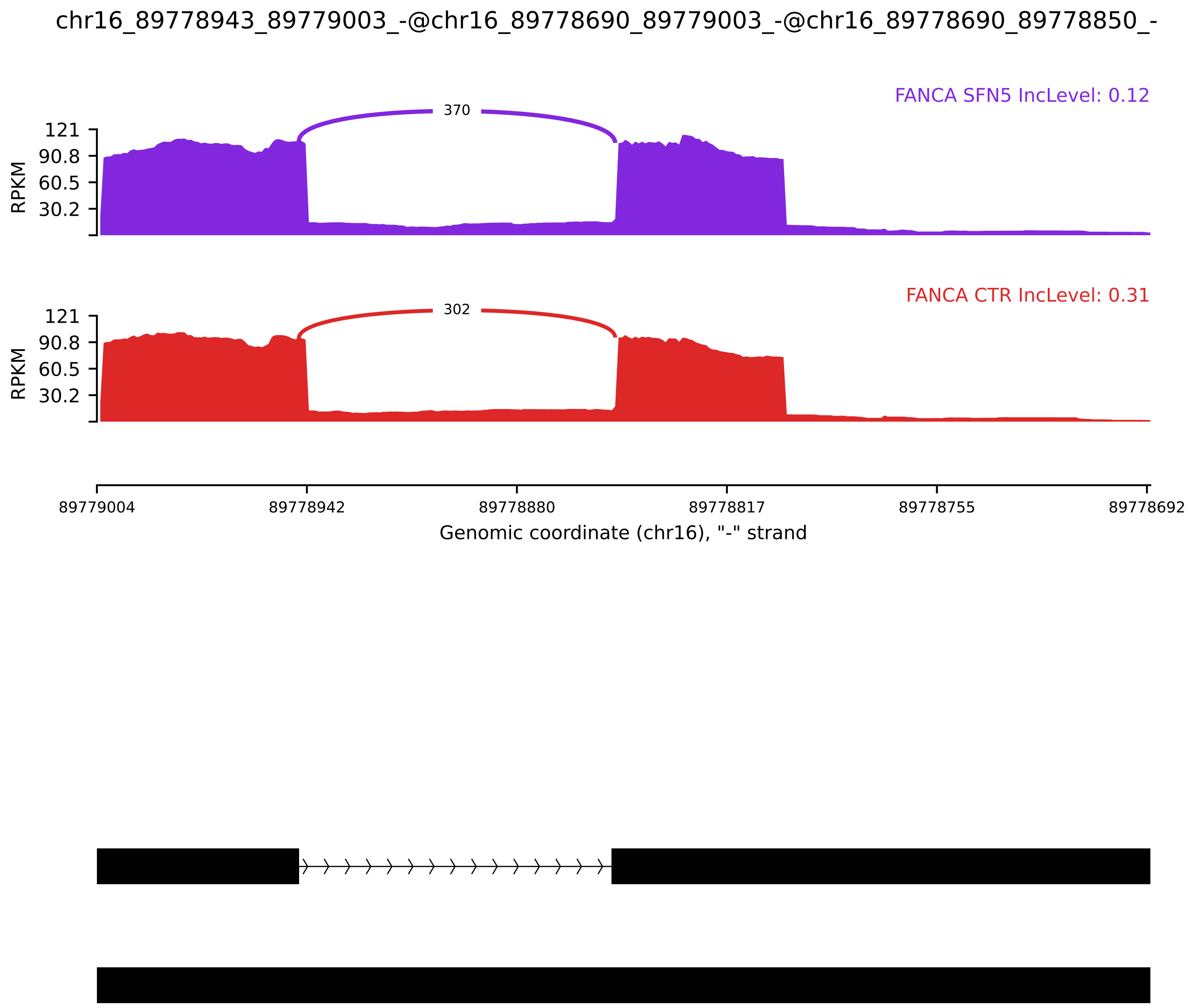

chr16_89778943_89779003_-@chr16_89778690_89779003_-@chr16_89778690_89778850_-
FANCA SFN5 IncLevel: 0.12
370
121
90.8
60.5
30.2
RPKM
FANCA CTR IncLevel: 0.31
302
121
90.8
60.5
30.2
RPKM
89779004
89778942
89778880
89778817
89778755
89778692
Genomic coordinate (chr16), "-" strand

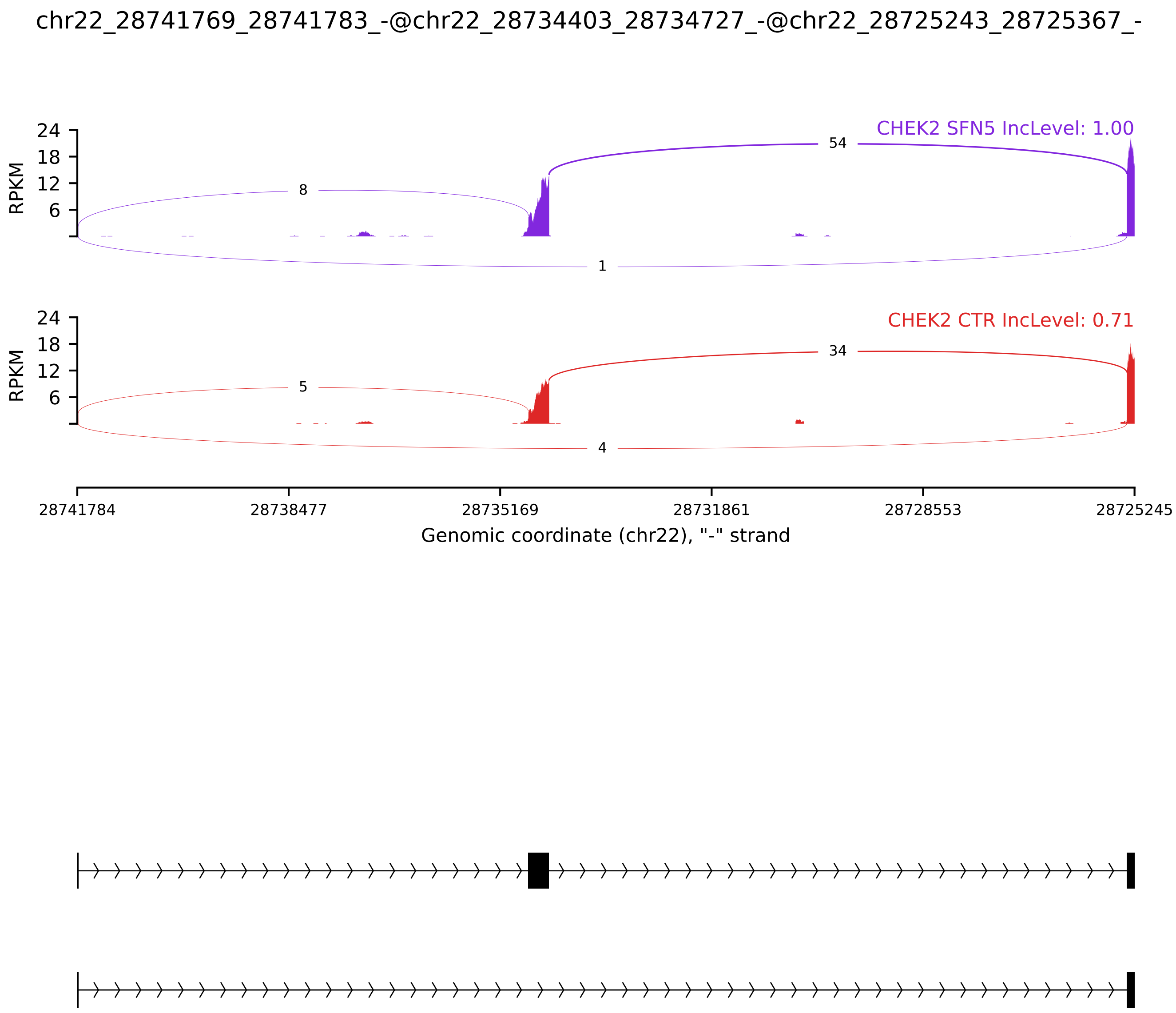

chr22_28741769_28741783_-@chr22_28734403_28734727_-@chr22_28725243_28725367_-
CHEK2 SFN5 IncLevel: 1.00
CHEK2 CTR IncLevel: 0.71
RPKM
24
18
12
6
54
8
1
34
5
4
28741784
28738477
28735169
28731861
28728553
28725245
Genomic coordinate (chr22), "-" strand

chr2_214797061_214797117_-@chr2_214792297_214792445_-@chr2_214780560_214781509_-

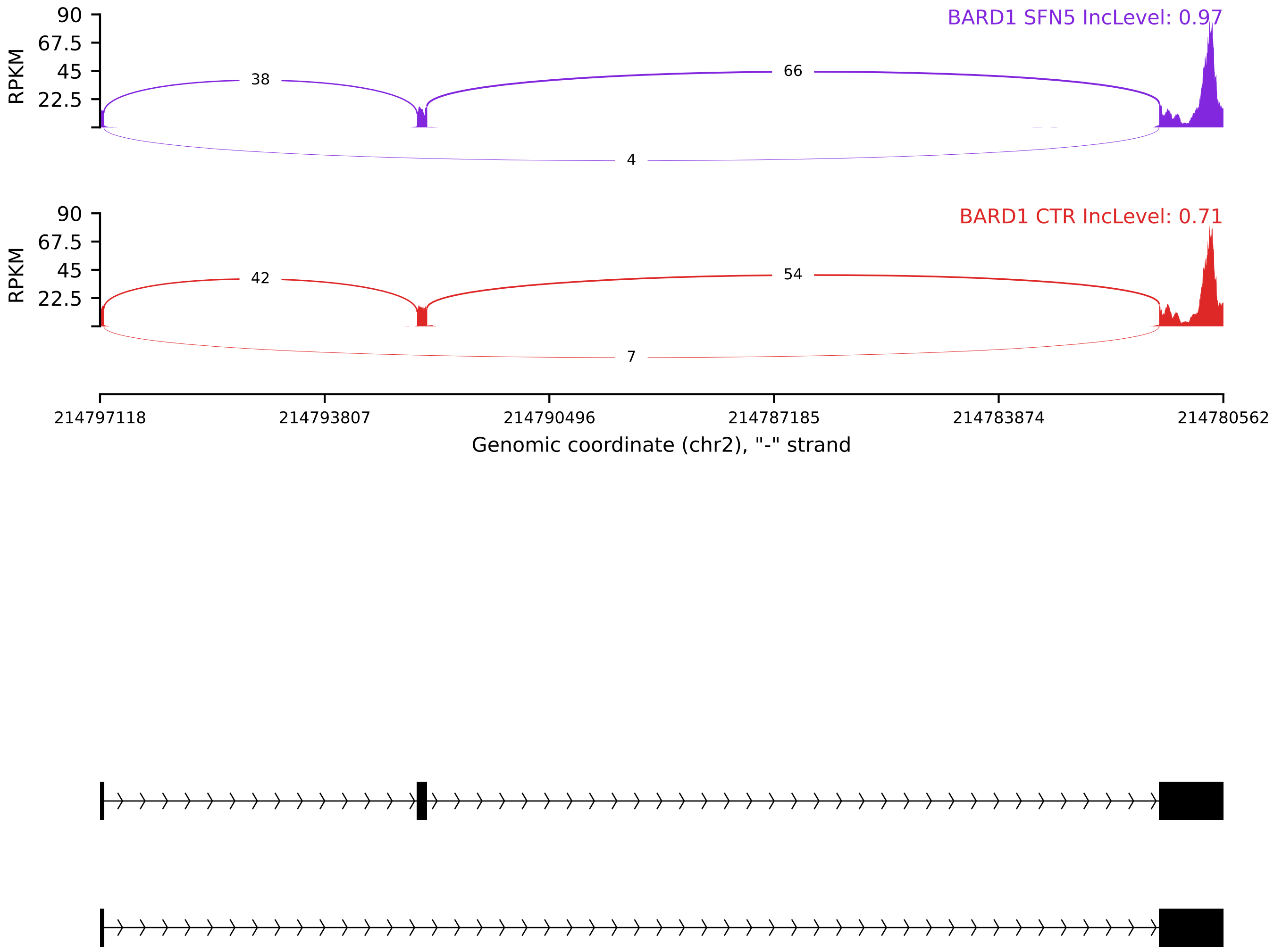

chr9_35078605_35078736_-@chr9_35078141_35078343_-@chr9_35077264_35077399_-

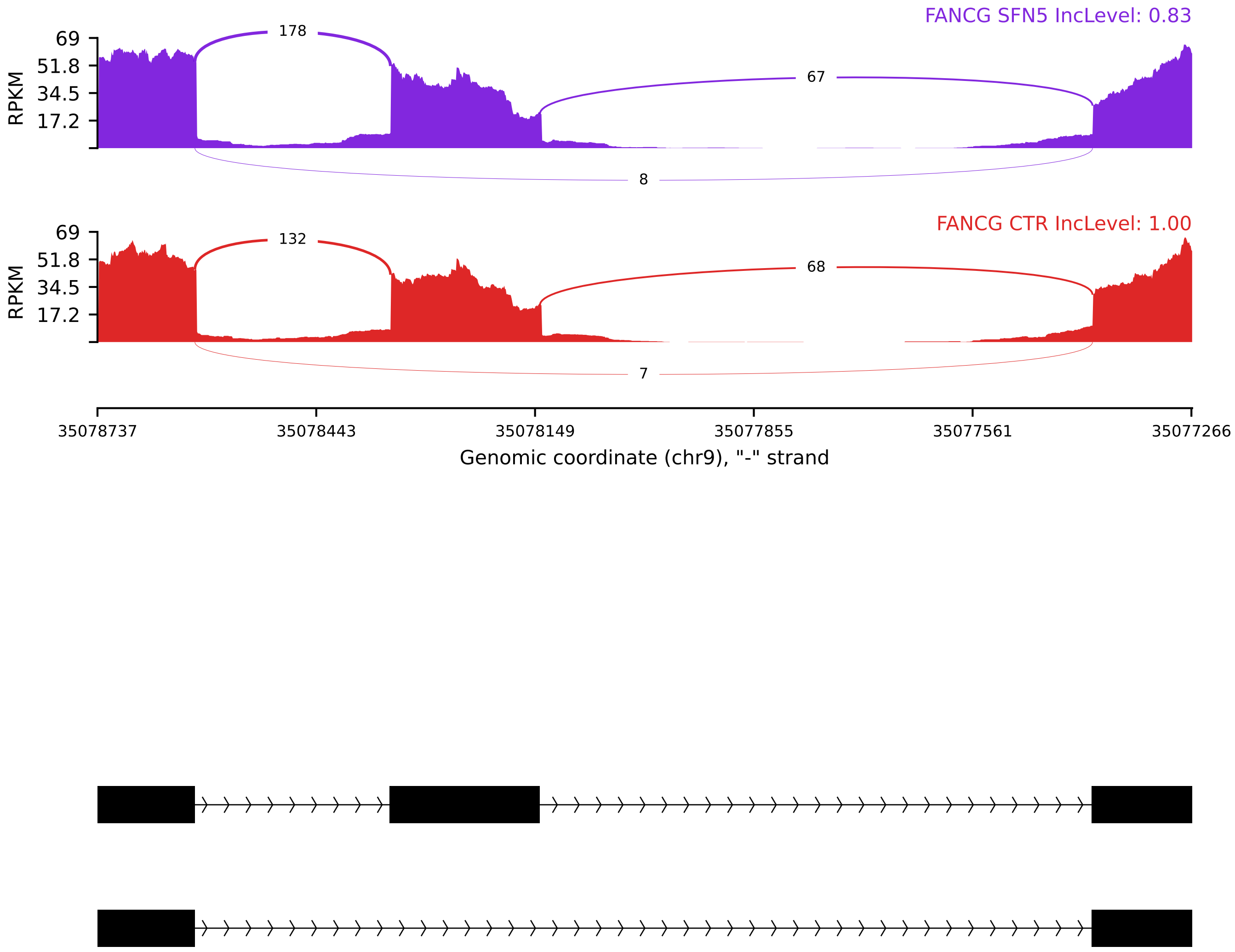

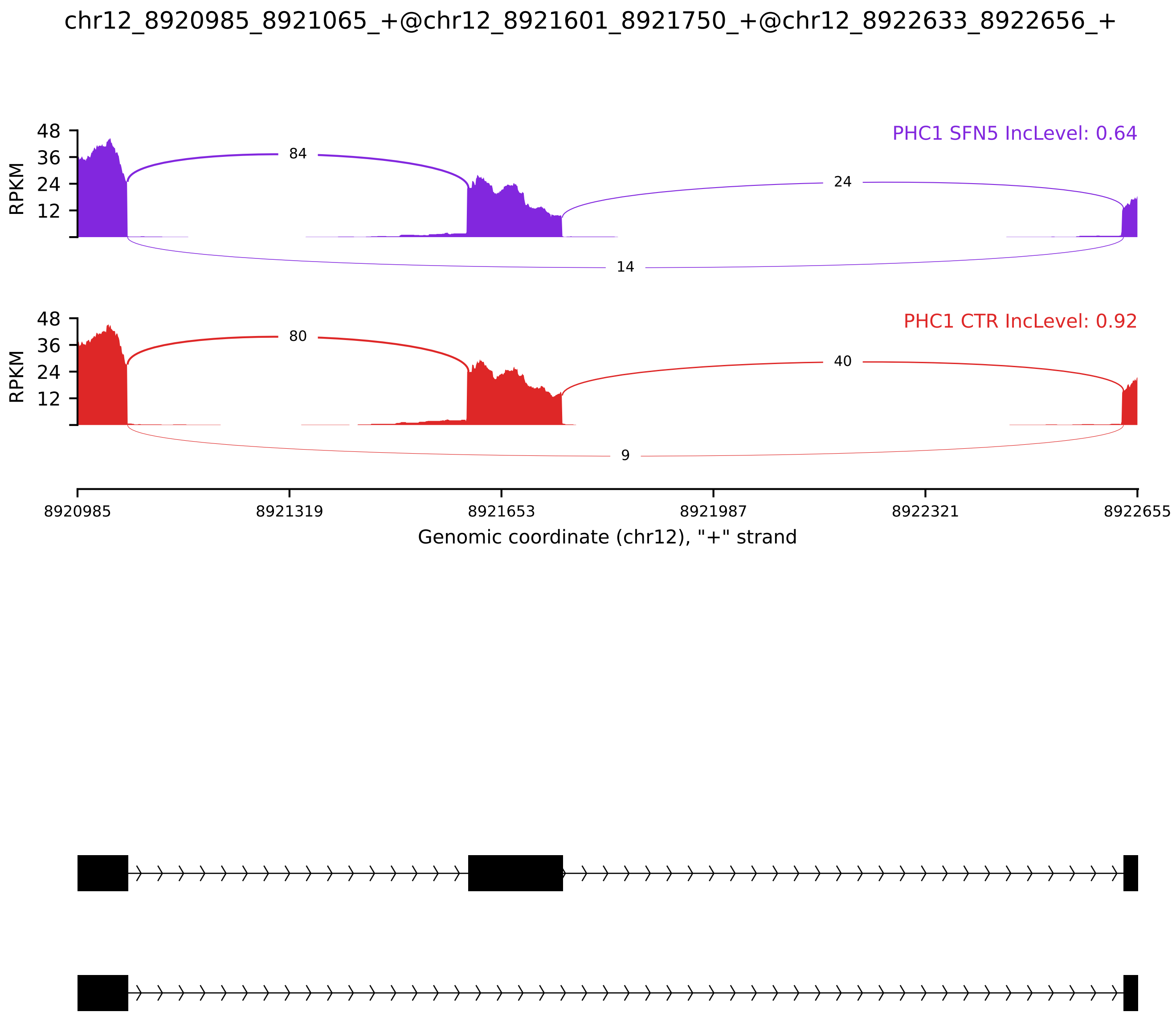
chr12_8920985_8921065_+@chr12_8921601_8921750_+@chr12_8922633_8922656_+
48
36
24
12
RPKM
84
24
14
PHC1 SFN5 IncLevel: 0.64
48
36
24
12
RPKM
80
40
9
PHC1 CTR IncLevel: 0.92
8920985
8921319
8921653
8921987
8922321
8922655
Genomic coordinate (chr12), "+" strand
