# Supplementary figures and images for: Computational Splicing Analysis of Transcriptomic Data Reveals Sulforaphane Modulation of Alternative mRNA Splicing of DNA Repair Genes in Differentiated SH-SY5Y Neurons
Source: Int J Mol Sci. 2025 Aug 23;26(17):8187. doi: 10.3390/ijms26178187 (PMC12428557; doi:10.3390/ijms26178187)

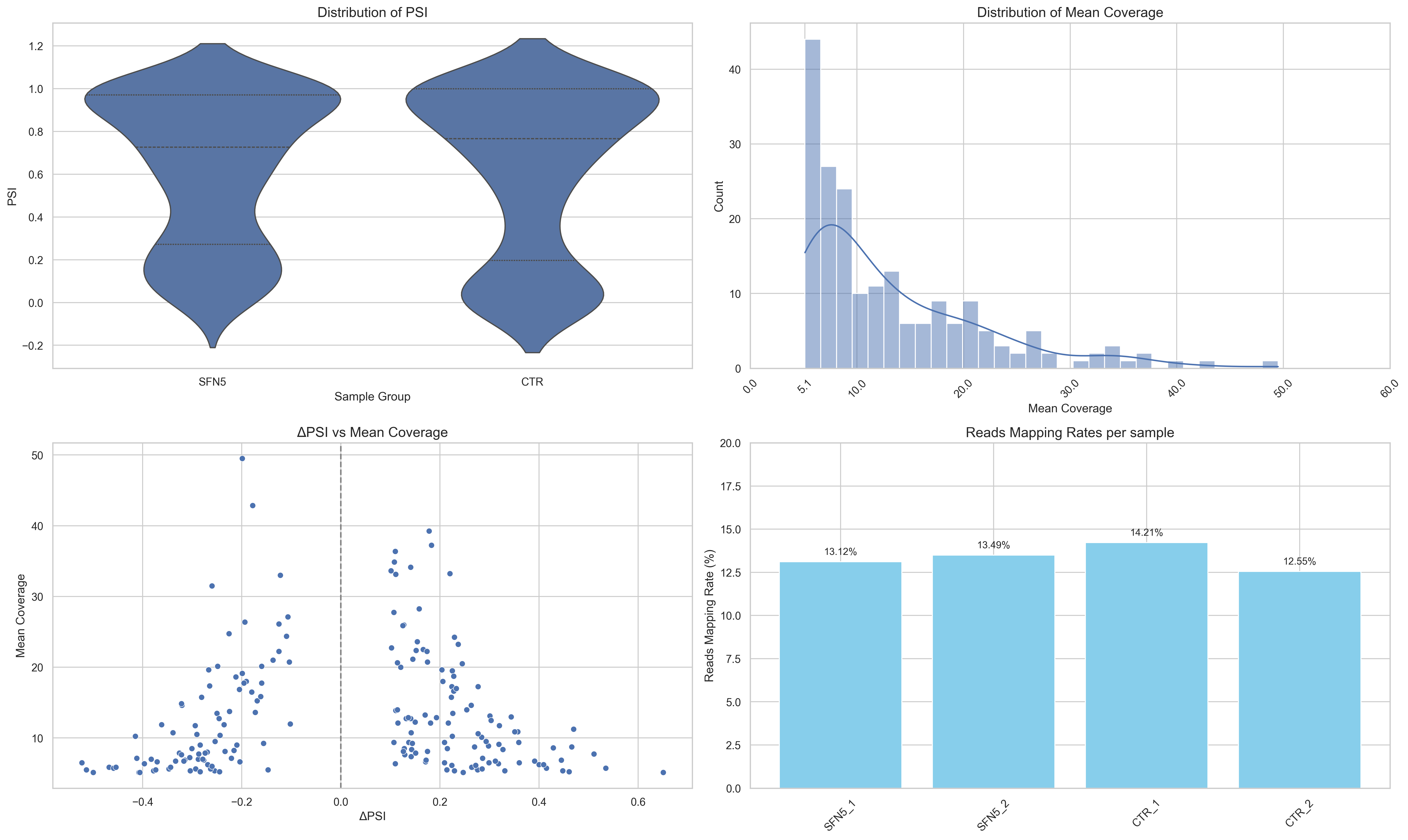

Supplement: Supplementary file 1 [file ijms-26-08187-s001.zip › Figure S1.png]
